# *GmFTIP09* regulated flowering time and seed weight

**DOI:** 10.3389/fpls.2025.1640116

**Published:** 2025-08-11

**Authors:** Lei Chen, Jiemin Chen, Chang Liu, Haiyang Li, Mengyang You, Qingshan Chen, Sijia Lu

**Affiliations:** ^1^ Guangdong Key Laboratory of Plant Adaptation and Molecular Design, Guangzhou Key Laboratory of Crop Gene Editing, Innovative Center of Molecular Genetics and Evolution, School of Life Sciences, Guangzhou University, Guangzhou, China; ^2^ School of Environmental Science and Engineering, Guangzhou University, Guangzhou, China; ^3^ Northeast Agricultural University, Harbin, China

**Keywords:** soybean, chromosome segment substitution lines, growth period, yield, *GmFTIP09*

## Abstract

Soybean is highly sensitive to photoperiod, which influences the growth period including flowering time (R1) and maturity (R8), ultimately affecting yield. In this study, we used a chromosome segment substitution lines population (CSSLs), generated by introgressing segments of *Glycine soja* ZYD00006 into *Glycine max* cultivar Suinong 14, to identify quantitative trait loci (QTL) associated with growth period and yield. A total of 130 QTLs were identified across three environments, including 88 QTLs for growth period and 42 QTLs for grain-related traits. Based on the distribution of these QTLs, we identified 16 QTL clusters across 12 chromosomes. Among these, Chr09-cluster-1 harbored three stable QTLs associated with R1, R8 and 100-seed weight (SW). The gene *GmFTIP09* was identified as the target gene. The mutant of *Gmftip09* delayed both flowering and maturity times, as well as reduced SW. The allele of early flowering, early maturity and large seed weight were under strict artificial selection during the early stage of modern soybean breeding. This research provides valuable insights into the genetic control of flowering time and seed traits, offering potential targets for soybean breeding.

## 1 Introduction

Soybean (*Glycine max* (L.) Merr.) is a primary source of dietary protein and vegetable oil (Hartman et al., 2011). Soybean yield is contributed by several traits, including growth period and seed weight. The reproductive stages (R), which affect the adaptability, productivity, and seed quality, are divided into eight stages (R1-R8) (Fehr et al., 1971). The R1 stage is defined as when the plant has one open flower on the main stem. The R2 stage occurs when an open flower is present at one of the two uppermost nodes on the main stem. The R3-R6 are associated with pod and seed development, including the R3 is defined as beginning pod, R4 means the full pod, R5 indicated the beginning seed and R6 means the full seed.R7 is defined as when one normal pod on the main stem has reached its mature pod color and R8 defined as when 95% of the pods have reached their mature pod color (Clay et al., 2013). Among these stages, R1 marking the transition from the vegetative phase to the reproductive phase, and the R8 means full maturity, and the interval from R1 to R8 (RP) represent the whole reproductive. All of these three traits are particularly important for maximizing soybean yield. Soybean grain yield is typical quantitative trait that is ultimately determined by the number of seeds per unit area and the seed weight (Pedersen and Lauer, 2004). The seed weight of soybean (often denoted by hundred seed weight) in cultivated soybean is not only an important target of breeders aiming to improve yield (Tayade et al., 2023), but also a trait that determines the grade of soybean and quality of soy-based foods (Friedman and Brandon, 2001).

The growth period and seed weight were all controlled by many genes with additive and epistatic effects (Gao et al., 2024). In previous studies, the biparental mapping populations were generally used for mapping quantitative trait loci (QTL). More than 600 genetic loci for growth period (including R1, RP, R8) and over 500 genetic interval for seed traits (including seed weight, seed weight per plant and see yield) were listed in SoyBase (https://www.soybase.org). Due to the inherently narrow genetic base of biparental populations, QTL detection efficiency is substantially reduced. As a high-throughput alternative to traditional linkage mapping, the genome wide association study (GWAS) achieves efficient QTL detection by correlating genome-wide single-nucleotide polymorphisms (SNPs) with phenotypic variation. Presently, GWAS have begun to effectively analyze the genetic basis of growth period and seed weight in soybean (Huang et al., 2010; Zhang et al., 2015, 2016; Yan et al., 2017; Zhao et al., 2019; Kim et al., 2020; Qi et al., 2020; Cao et al., 2022; Yao and Zhang, 2024; Perfilev et al., 2024). However, both of the two ways can only map association signals to large genomic regions, with the complicated genetic backgrounds, making it hard to identify the candidate genes. Chromosomal segment substitution lines (CSSLs), each carrying one or a few specific marker-defined donor segments in the genetic background of the adapted cultivar (Surapaneni et al., 2017), not only can improve the accuracy of QTL mapping (Ando et al., 2008; Ookawa et al., 2016), but also can be directly used in breeding by design. Therefore, CSSLs serve as a valuable platform for breeding by design through target chromosome segment substitutions (Zhu et al., 2009; Li et al., 2019; Zhang et al., 2021).

Domestication was the earliest form of plant breeding and played an essential role in the rising of agriculture (Diamond, 2002; Salamini et al., 2002; Doebley et al., 2006). Modern crops differ from their wild relatives due to changes in various agronomically important traits that collectively referred to as the domestication syndrome, including the loss of seed dormancy and pod shattering, a decrease in branching, an increase in fruit or seed dimensions, modifications in photoperiod sensitivity, and the advancement and synchronization of flowering and maturation processes (Darwin, 1859). It is widely believed that cultivated soybean was domesticated from wild soybean (*Glycine soja* Sieb. & Zucc.) in China approximately 6000–9000 years ago (Doebley et al., 2006; Kim et al., 2010; Wang and Li, 2011). Compared with wild soybean, cultivars showed earlier flowering time and larger seed weight (Lu et al., 2020; Goettel et al., 2022; Li et al., 2024b), but very few domestication-related genes regulating this trait are currently known, such as *Tof12* for early flowering and maturity, *POWR1* and *GmCYP82C4* for seed weight. The selection of the domesticated traits resulted in a genome-wide reduction of genetic diversity, as well as loss of useful traits reserved in wild relatives. During the domestication, severe genetic bottlenecks occurred, resulting in the loss of more than 70% of rare alleles, which highlights the high allelic diversity in wild soybeans (Hyten et al., 2006; Zhuang et al., 2022). Unraveling the domestication genetics of soybean will facilitate the discovery and utilization of rare but potentially important alleles.

To further identify key genes regulating soybean flowering time and seed weight during domestication, and to exploit superior genetic resources from wild soybean, this study employed a chromosome segment substitution line (CSSL) population, with wild soybean as the donor parent and cultivated soybean as the receptive parent. By integrating phenotypic data collected over three years, we identified 88 quantitative trait loci (QTLs) related to growth period, 42 QTLs associated with seed-related traits. The results revealed distinct regulatory mechanisms for flowering time and maturity, with the post-flowering stage playing a significant role in soybean yield. Additionally, we also detected 16 QTL clusters, and the candidate gene within the Chr09-cluster-1, the most stable cluster, was analyzed. Interestingly, no previously reported loci associated with flowering time or seed weight were found within this region. Results clarified that *GmFTIP09* may be the candidate gene regulating both soybean flowering time and 100-seed weight. Notably, the early-flowering haplotype of *GmFTIP09* underwent strong selective pressure during domestication. These findings provide valuable genetic resources for understanding the regulation of soybean growth periods and improving crop yield.

## 2 Materials and methods

### 2.1 Plant materials and phenotypic analysis

QTL Detection: The seeds of the CSSL population, which consisted of 170 lines constructed from ZYD00006 (ZY06, donor parent) and Suinong 14 (SN14, recurrent parent), were provided by the Northeast Agricultural University (Xin et al., 2016). In 2017, 2018 and 2019, the population of CSSLs were cultivated in experimental fields in Harbin, China (45°75′N, 126°63′E), where features Mollisols. Sowing operations were conducted between May and October. Every soybean material was cultivated in 2-meter-long rows, with 60 cm inter-row spacing. 20 plants were sown per row. Five plants per row were randomly sampled to record R1(the first flower that appeared on 50% of the plants), R8 (the pods of 50% of the plants that had a mature color), RP (the interval from R1 to R8), PM (the percentage of RP within the R8), SW (100-seed weight per plant) and GW (all seed weight per plant), then the line means used four subsequent analyses. In addition, SN14 and CSSL77 were grown in growth chambers under long-day photoperiods (16 h light/8 h dark) for sample collection and RT-PCR assays.

Phenotypic screening of mutant libraries: The Willimas 82 (Wm82) and mutant library induced by ethyl methanesulfonate (EMS) were provided by the Nanjing Agricultural University (Zhang et al., 2022). Wm82 and mutants were sown in Ningxia, China (38°23′N, 106°23′E), where is dominated by Fluvo-Aquic soils. Sowing operations begins in May and harvested in October, 2023. Every line was cultivated in 2-meter-long rows, with 60 cm inter-row spacing. 20 plants were sown per row. Five plants per row were randomly sampled to record R1, R8, and SW. In addition, Wm82 and homozygous *Gmfip09–1* EMS mutants were grown in growth chambers under long-day photoperiods (16 h light/8 h dark) to further characterize the phenotype.

The QTLNetwork software was employed to estimate the heritability of traits (Yang et al., 2008).

The three-year phenotypic mean used for collinearity analysis, and the R software was carried out for correlation analysis and visualization.

The statistical analysis and visualization were performed in SPSS and GraphPad Prism, respectively.

### 2.2 Detection of introgressive chromosome segments

To investigate the SNP markers, CSSLs of the population were re-sequenced using the Illumina Hiseq 2000 platform (20x coverage), a total of 3895 high-quality polymorphic bin marker were distributed on 20 chromosomes. Based on the physical map constructed in this study, markers evenly distributed on the physical map were selected to genotype the 170 individuals of CSSL population. GGT2.0 software (Van Berloo, 2008) was applied to analyze the characteristics of chromosomal introgressed segments (background recovery rate of the CSSLs, the number and length of introgressed segments) with default parameters.

### 2.3 Identification of maturity- and grain-related QTL

MapQTL 6.0 was applied to map QTLs by Multiple-QTL model (MQM), with a threshold of LOD≥2.0 (Van Ooijen, 2009). Positive additive effects indicated that ZY06 contributed to the favorable alleles of QTLs, whereas negative additive effects indicated SN14 contributed to the favorable alleles. QTLs were named according to their trait and the order on the chromosome. A QTL cluster was defined when a chromosomal region contained three or more QTLs for different traits, and their confidence intervals shared an overlapping genomic segment (Yang et al., 2022).

### 2.4 Sequence alignment and phylogenetic analysis

The *AtFTIP1* (*AT2G45660*), *AtFTIP3* (*AT3G57880*), *AtFTIP4* (*AT1G51570*), *AtFLD* (*AT3G10390*) and *AtFLD-Like* (*AT1G62830*) protein sequence from *Arabidopsis* was retrieved from TAIR (https://www.arabidopsis.org/). The *FTIP* and *FLD* proteins sequences from *Glycine max* are available at Phytozome (https://phytozome-next.jgi.doe.gov). Multiple sequence alignment was performed with Muscle in MEGA version 7.0 using default parameters. A neighbor-joining (NJ) phylogenetic tree was constructed with MEGA 7.0. The amino acid sequences were aligned and shaded with DNAMAN software.

### 2.5 RNA extraction and RT-qPCR

The trifoliate leaves of SN14, CSSL77 were sampled at 20 DAE at Zeitgeber time (ZT) 16 in long-day conditions (16 h light/8 h dark) for expression analysis. Total RNA was extracted from samples using an Ultrapure RNA Kit (CWBIO, Jiangsu, China). First-strand cDNA synthesis and removal of genomic DNA contaminants were performed using a HiScript^®^ III RT SuperMix for qPCR (+gDNA wiper) (Vazyme, Nanjing, China). Quantitative PCR (qPCR) was performed using a Roche LightCycle480 system (Roche, Mannheim, Germany) using a qPCR kit (Roche). *β-Tubulin* (*TUB*, *Glyma.05g157300*) was used as the internal control. Three independent biological replicates were analyzed, and three technical replicate reactions were used for each sample. The statistical analysis and visualization were performed in SPSS and GraphPad_Prism, respectively. All qPCR primers are listed in **Supplementary Table S1**.

## 3 Results

### 3.1 Introgressive segments analysis of the CSSLs

A chromosome segment substitution lines (CSSLs) population was constructed in a previous study (Xin et al., 2016). However, the limited number of molecular markers available in this population restrict the efficiency of the candidate gene discovery. To address this, we re-sequenced the population using the Illumina Hiseq 2000 platform (20x coverage) and identified 3895 high-quality polymorphic bin markers that distributed across all 20 chromosomes.

**Figure 1:**
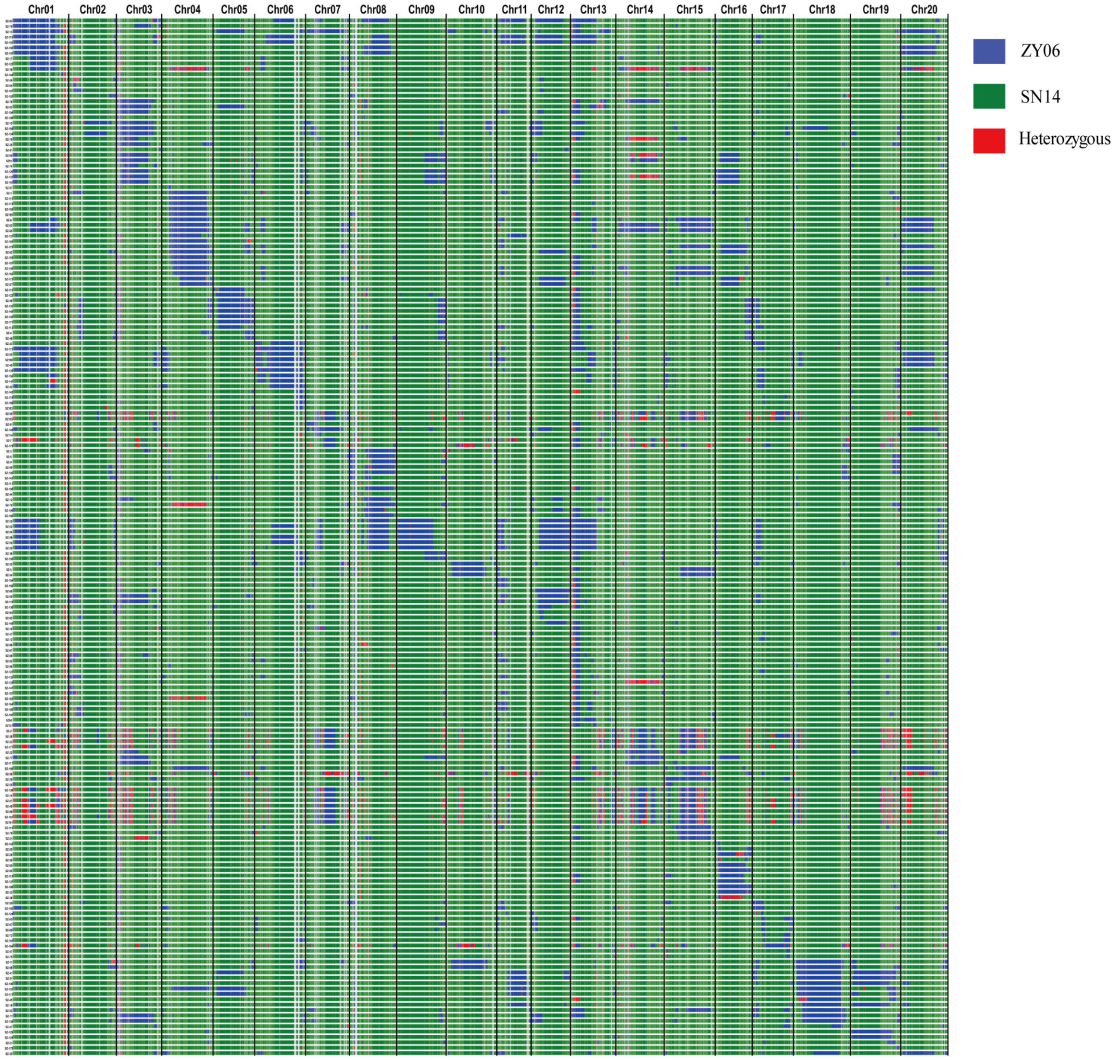
Genetic constitution and introgressive segments of CSSLs. Distribution of introgressed segments in the CSSLs on the 20 chromosomes. Blue: Homozygous for the allele from donor parent ZY06; Green: Homozygous for the allele from donor parent SN14; Red: Heterozygous.

As shown in **Figure 1**, the introgressed fragment spanned the set of 20 chromosomes. The proportion of the introgressed region relative to each chromosome ranged from 1.20% to 23.80%, with an average of 8.87% (**Supplementary Figure S1A**). For each CSSL, the number of introgressed fragment ranged from 30 to 105 (**Supplementary Figure S1B**), and the physical distance of introgressed fragment ranged from 5.60 to 214.77 Mb, with an average of 74.58 Mb (**Supplementary Figure S1C**). Large gaps were observed on chromosomes 2, 6, 9, 12, 17, and 19, indicating the uneven distribution of markers across chromosomes. These gaps may reflect significant variations in recombination rates at different chromosomal locations.

### 3.2 Characterization of phenotypic performance

All traits exhibited fluctuations across different years (**Supplementary Figure S2**). The interannual variation of flowering time (R1) was smaller, from 29.72 to 57.66 days, than that of the maturity (R8), from 94.66 to 136 days. Both R8 and RP (the interval from R1 to R8) showed similar interannual trends that 2019 was the highest, followed by 2017 and 2018. However, the interannual trend of R1 was different, with 2017 being the highest, followed by 2018 and 2019. This divergence suggests that distinct regulatory mechanisms may control R1 and R8. 100 seed weight per plant (SW) and seed weight per plant (GW) ranged from 8.59 g to 27.52 g and 5.81 g to 28.76 g, respectively. We conducted a comparative analysis of six traits and found that R1, R8, and SW are mainly influenced by genetic factors, while the other three traits are more significantly affected by environmental factors (**Supplementary Table S2**).

**Figure 2:**
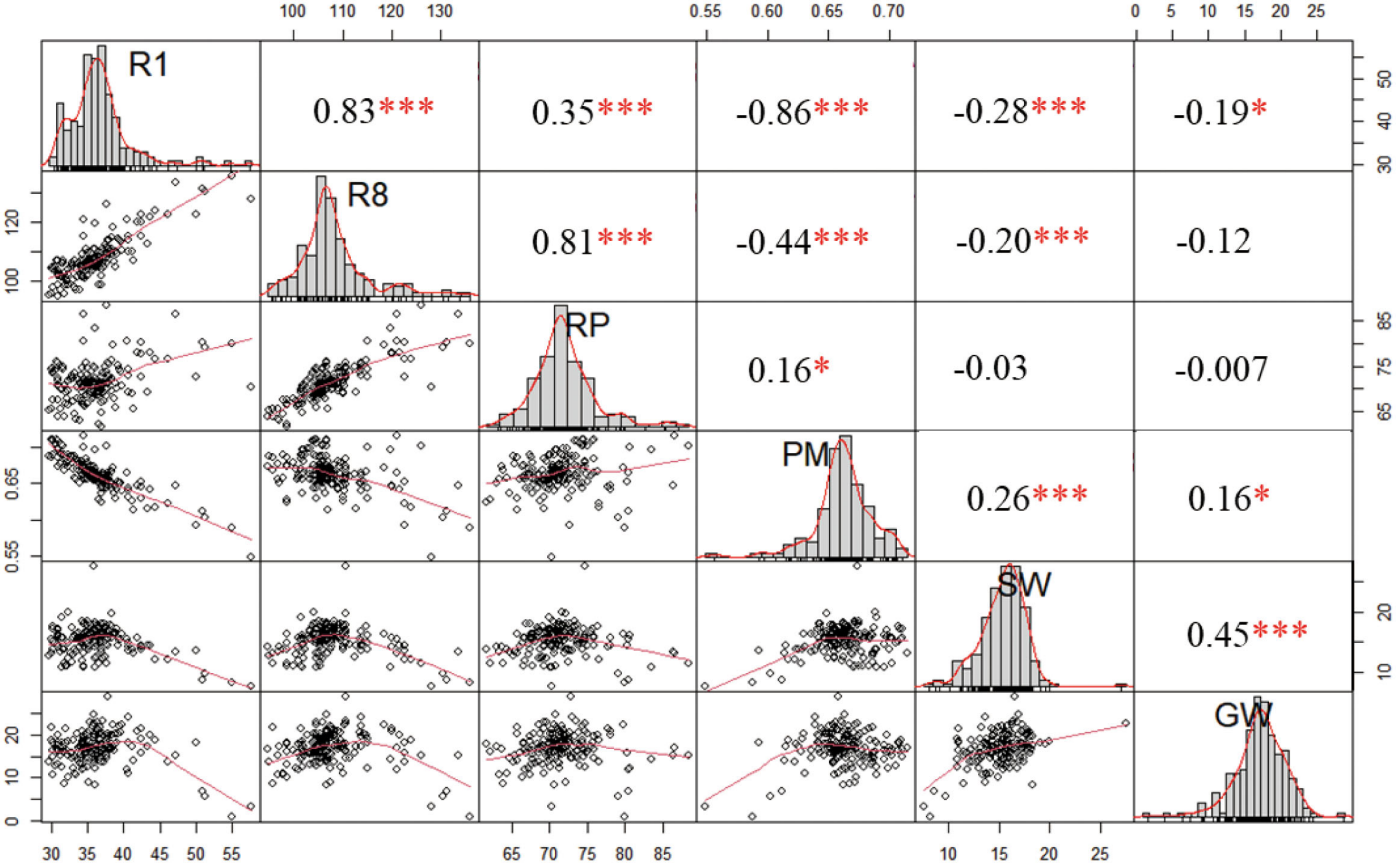
Correlation analysis among maturity- and grain-related traits. The values in the correlation matrix represent Pearson´s correlation coefficient. Positive value represents positive correlation and negative value represents negative correlation. * and *** represent significant differences at the 0.05 and 0.001 probability levels, respectively. R1: Flowering time (day); R8: Maturity (day); RP: The interval from R1 to R8 (day); PM: The percentage of RP within the R8 (%); SW: 100-seed weight (g); GW: Grain weight per plant (g).

Correlation analysis of all traits revealed a significant positive correlation between GW was with SW, though the correlation coefficient was relatively low (**Figure 2**). This suggested that additional factors, beyond SW, are involved in regulating GW. Interestingly, we found a negative correlation between GW and R1, but a positive correlation between GW and the proportion of RP within R8 (PM) (**Figure 2**). These results imply that the long vegetative photoperiod may not be favorable for yield, whereas an optimal PM is a critical factor in determining yield.

### 3.3 QTL mapping for growth period and grain traits

A total of 130 quantitative trait loci (QTLs) were detected, which were randomly distributed across 20 chromosomes. Among them, the fewest (only one) QTL was detected on chromosomes 8 and 9, the most (twelve) QTLs were located on chromosomes 6. The majority of QTLs were distributed at both ends of chromosomes, which could be attributed to the fact that chromosomes telomeres is typically hotspots for recombination.

**Figure 3:**
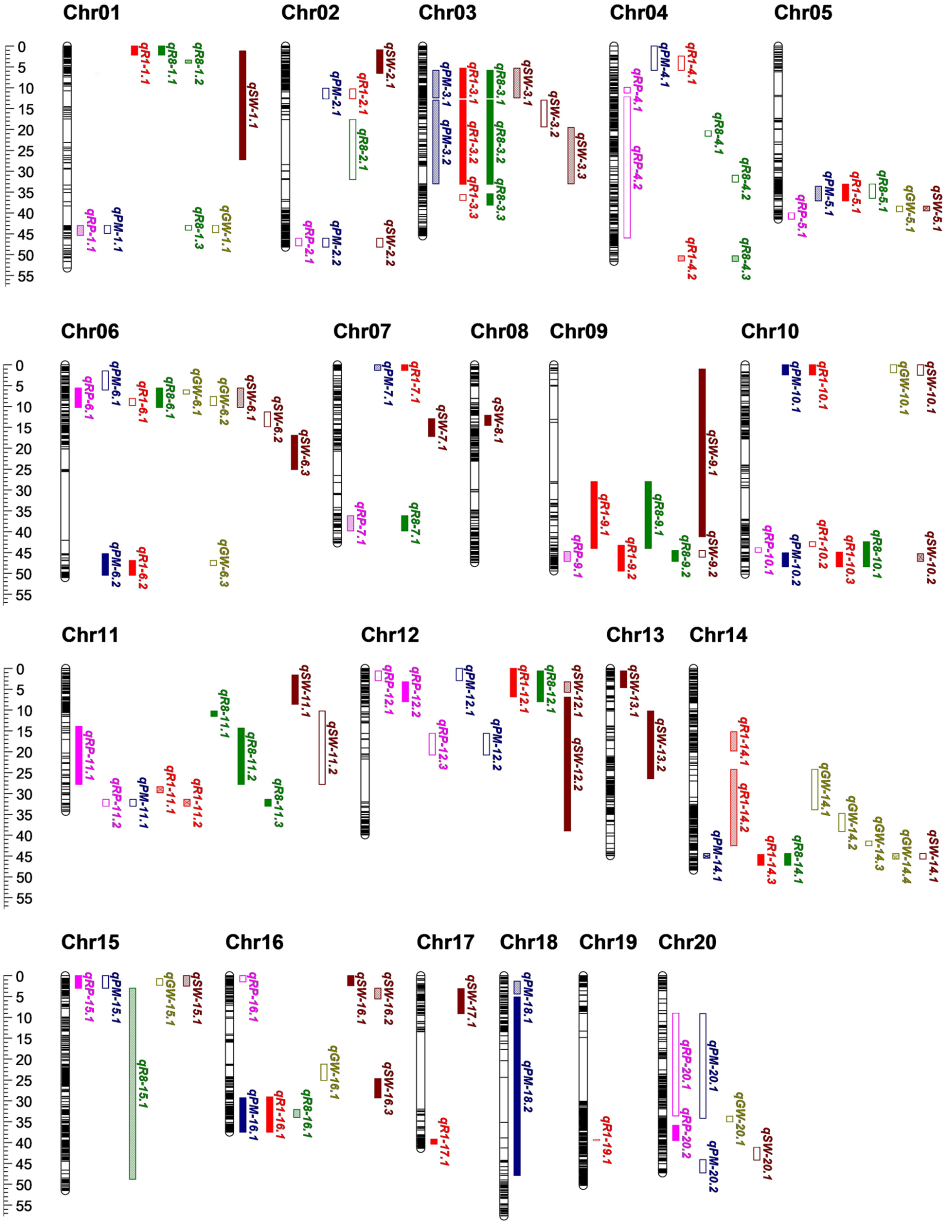
QTLs detected on all chromosomes. Red, green, purple, blue, brown, yellow fonts represent R1, R8, RP, PM, SW and GW, respectively. Outlined box indicate that QTL was detected in one environment; an outlined box that filled with a grid of outlined diamonds indicate that QTL was detected in two environments; solid box indicate that QTL were detected in three environments, respectively.

Of the 130 QTLs, 88 were associated with the growth period (**Figure 3**; **Supplementary Table S3**), including 25 QTLs for R1, 23 QTLs for R8, 18 QTLs for RP, and 22 QTLs for PM. Among these, 18 QTLs were detected in two environments, and 38 QTLs were detected in three environments, all of which are considered stable QTLs. Almost all of the R1 and R8 QTLs were stable, indicating the strong genetic control over these traits. For R1 and R8, the additive effect of most QTLs was positive, meaning that the allele derived from SN14 resulted in earlier flowering and maturation. However, around the marker Block3364, one locus controlling the R1, R8 and RP simultaneously, allelic variations from the wild soybean ZY06 at this locus promote earlier flowering and maturation. This result indicated that there are also early flowering and maturing locus in late flowering wild soybeans. Compared with R1 and R8, the additive effect of the RP showed a different trend, with half of the QTLs having positive effects and the others half negative. Additionally, we also found that QTL near the markers Block4028 regulated RP and R8, but not R1, and QTL near the marker Block8903 regulated R1 and R8, but not RP. These findings suggested the molecular mechanisms underlying R1, R8 and RP may differ.

Furthermore, 42 QTLs associated with seed traits were identified (**Figure 3**; **Supplementary Table S3**), including 13 QTLs for seed weight per plant (GW) and 29 QTLs for 100 seed weight per plant (SW). Most of the SW QTLs were stable, being detectable in two or three environments, indicating that genetic factors significantly influence SW. In contrast, GW was influenced by more factors, making it more complex. Most of the GW QTLs were only detected in one environment, with the exception of the *qGW-14.4*, which also regulated the SW and may present a major QTL for seed traits in CSSLs. The allelic variation of SN14 is favorable for increasing both SW and GW.

Based on the distribution of the QTLs, we identified 16 QTL clusters across 12 chromosomes (**Table 1**). Among these, one (Chr02-cluster-1) contained no stable QTL, five contained one stable QTL, and ten contained more than two stable QTLs. Growth was consistently linked to the seed traits. We found that 13 of the clusters regulated both growth period and grain-related traits, indicating a coordinated genetic control of these two traits. However, Chr05-cluster-1, Chr11-cluster-2 and Chr16-cluster-1 regulated only growth period, suggesting that the candidate genes for these loci could be used to improve ecological adaptability without compromising yield.

**Table 1:** QTL clusters identified in the CSSLs across multiple environments.

Cluster	QTLs
Chr01-cluster-1	*qRP-1.1* ^(-)^ *, qPM-1.1* ^(-)^ *, qR8-1.3* ^(-)^ *, qGW-1.1* ^(-)^
Chr02-cluster-1	*qRP-2.1* ^(+)^ *, qPM-2.2* ^(-)^ *, qSW-2.2* ^(-)^
Chr03-cluster-1	*qPM-3.1* ^(-)^ *, qR1-3.1* ^(+)^ *, qR8-3.1* ^(+)^ *, qSW-3.1* ^(-)^
Chr03-cluster-2	*qPM-3.2* ^(-)^ *, qR1-3.2* ^(+)^ *, qR8-3.2* ^(+)^ *, qSW-3.2* ^(-)^ *, qSW-3.3* ^(-)^
Chr05-cluster-1	*qPM-5.1* ^(+)^ *, qR1-5.1* ^(-)^ *, qR8-5.1* ^(-)^
Chr06-cluster-1	*qRP-6.1* ^(-)^ *, qR1-6.1* ^(-)^ *, qR8-6.1* ^(-)^ *, qGW-6.1* ^(-)^ *, qGW-6.2* ^(-)^ *, qSW-6.1* ^(-)^
Chr09-cluster-1	*qR1-9.1* ^(+)^ *, qR8-9.1* ^(+)^ *, qSW-9.1* ^(-)^
Chr09-cluster-2	*qRP-9.1* ^(+)^ *, qR1-9.2* ^(+)^ *, qR8-9.2* ^(+)^ *, qSW-9.2* ^(-)^
Chr10-cluster-1	*qPM-10.1* ^(-)^ *, qR1-10.1* ^(+)^ *, qGW-10.1* ^(-)^ *, qSW-10.1* ^(-)^
Chr10-cluster-2	*qPM-10.2* ^(-)^ *, qR1-10.3* ^(+)^ *, qR8-10.1* ^(+)^ *, qSW-10.2* ^(-)^
Chr11-cluster-1	*qRP-11.1* ^(+)^ *, qR8-11.2* ^(+)^ *, qSW-11.2* ^(-)^
Chr11-cluster-2	*qRP-11.2* ^(+)^ *, qPM-11.1* ^(-)^ *, qR1-11.2* ^(+)^ *, qR8-11.3* ^(+)^
Chr12-cluster-1	*qRP-12.2* ^(+)^ *, qR1-12.1* ^(+)^ *, qR8-12.1* ^(+)^ *, qSW-12.1* ^(-)^
Chr14-cluster-1	*qPM-14.1* ^(-)^ *, qR1-14.3* ^(+)^ *, qR8-14.1* ^(+)^ *, qGW-14.4* ^(-)^ *, qSW-14.1* ^(-)^
Chr15-cluster-1	*qRP-15.1* ^(-)^ *, qPM-15.1* ^(-)^ *, qGW-15.1* ^(-)^ *, qSW-15.1* ^(-)^
Chr16-cluster-1	*qPM-16.1* ^(+)^ *, qR1-16.1* ^(-)^ *, qR8-16.1* ^(-)^

^(+)^: indicate that ZY06 alleles increased the phenotypic value.

^(-)^: indicate that SN14 alleles increased the phenotypic value.

: indicate that the QTL can detected in three environments.

Of the identified clusters, the additive effect for growth period was always positive, while the additive effect for seed trait was negative, namely the favorable alleles from SN14 promoted flowering time and the optimized grain yield. This aligns with the goal of crop selection for early flowering, which facilitates mechanized harvesting, and high yield. However, Chr01-cluster-1 and Chr06-cluster-1 showed different patterns: the additive effects for both the growth period and seed trait QTLs were the same, meaning that the alleles from SN14 delay flowering time with higher SW and GW.

Among all the clusters, Chr09-cluster-1 is particularly noteworthy. All QTLs in this cluster were detected across three environments, emphasizing importance of identifying the candidate genes within this cluster. These genes would provide valuable loci for further functional research and soybean breeding. Therefore, we will focus on characterizing the candidate genes for this cluster in further studies.

### 3.4 Candidate genes prediction in Chr09-cluster-1

**Figure 4:**
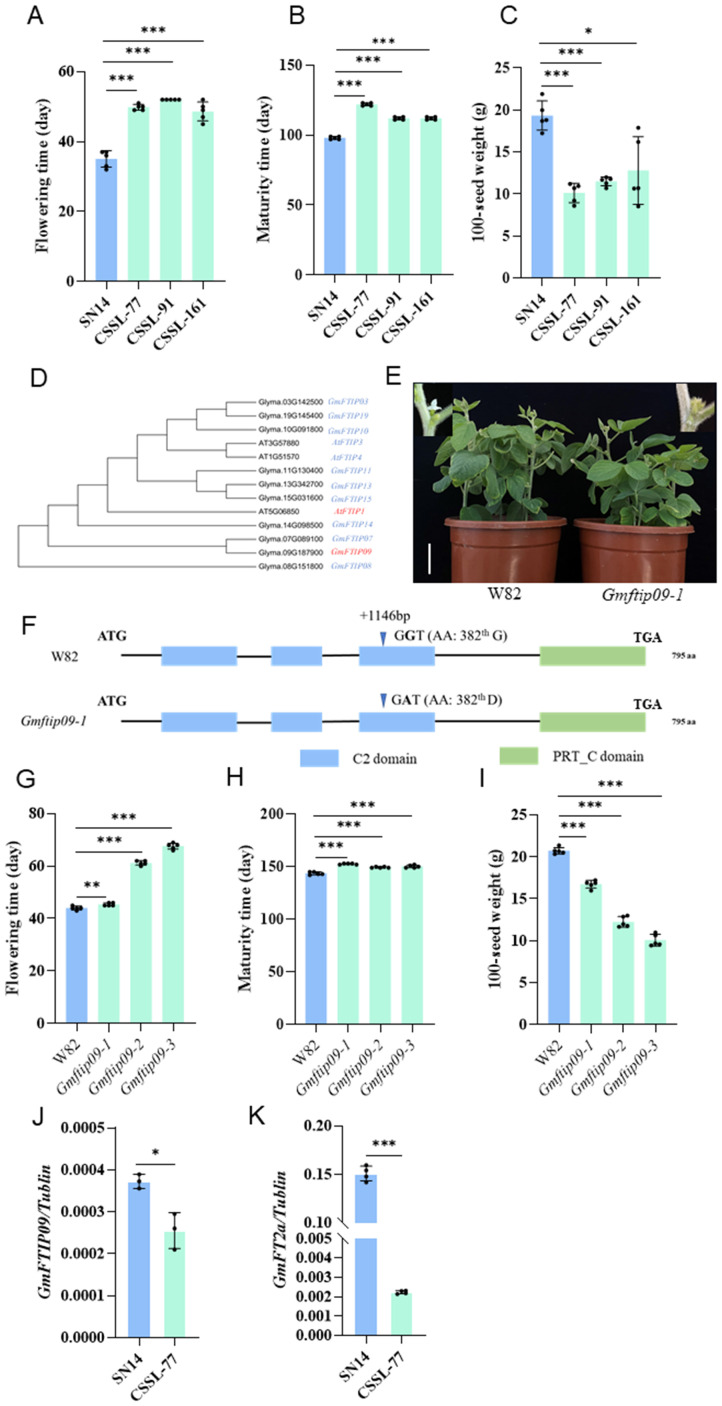
Candidate genes prediction in Chr09-cluster-1. **(A-C)** The phenotypic performance of SN14 and three CSSL lines carrying introgressed segments from Chr09-cluster-1 in Harbin. **(A)** Flowering time (R1); **(B)** Maturity (R8); **(C)** 100-seed weight (SW). All data are means ± SD (n=5). **(D)** Phylogenetic tree based on the amino acid sequences of FTIP proteins and their homologous proteins using the neighbor-joining method. **(E)** Phenotypes of wild-type plants (WT, W82) and homozygous *Gmftip09* EMS mutants in growth chambers under long-day photoperiods (16 h light/8 h dark). Scale bar, 5 cm. **(F)** Gene structures of the *GmFTIP09* show in W82 and *Gmftip09-1*. +, coding regions (CDS). AA, Amino acid. The blue bars represent C2 domain, green bars represent PRT_C domain. The triangle symbol represents the base mutation position. **(G-I)** The phenotypic performance of *Gmftip09* EMS mutant in Ningxia. The EMS mutant of *Gmftip09* has three different mutation types, named *Gmftip09-1*, *Gmftip09-2*, and *Gmftip09-3*, respectively. Compared with Wm82, *Gmftip09–1* harbors one non-synonymous variations at the coding sequence (CDS) positions of 1146 and caused a conversion of the 382^th^ amino acid from glycine to aspartic acid **(G-D)**; *Gmftip09–2* harbors one non-synonymous variations at the CDS positions of 1714 and caused a conversion of the 572^th^ amino acid from glutamic acid to lysine **(E-K)**; *Gmftip09-3*, harbors one non-synonymous variations at the CDS positions of 1822 and caused a conversion of the 608^th^ amino acid from alanine to threonine **(A-T)**. **(G)** Flowering time (R1); **(H)** Maturity (R8); **(I)** 100-seed weight (SW). All data are means ± SD (n=5). **(J, K)** Relative expression levels of *GmFTIP09* **(J)** and *GmFT2a* **(K)** in SN14 and CSSL-77 at ZT16. Data shown in are relative to the control gene *Tubulin*. Data shown are means ± SD from three independent biological replicates. All plants were grown in growth chambers under long-day photoperiods (16 h light/8 h dark). The two-sided Student’s t-test was performed to determine statistically significant differences in **(A-C)** and **(G-K)** *, ** and *** represent significant differences at the 0.05, 0.01 and 0.001 probability levels, respectively.

Focusing on Chr09-cluster-1, we grew SN14 and three CSSL lines (CSSL-77, CSSL-91, CSSL-161), which carrying introgressed segments from Chr09-cluster-1, in Harbin to analyze their phenotype. Compared to SN14, the three lines exhibited delayed flowering and maturity, as well as reduced SW. This indicates that the segment contains key genes regulating R1, R8 and SW (**Figures 4A-C**).

According to the Wm82 soybean reference genome from the Phytozome database, Chr09-cluster-1 contains six genes homologous to flowering-related genes in *Arabidopsis*, including *Glyma.09G143500*, the ortholog of *Arabidopsis TERMINAL FLOWER 1* (*TFL1*)*, Glyma.09G149000*, the ortholog of *Arabidopsis AGAMOUS-LIKE 6* (*AGL6*)*, Glyma.09G161300*, the ortholog of *Arabidopsis POLYMERASE-ASSOCIATED FACTOR 2* (*PAF2*)*, Glyma.09G185800*, the ortholog of *Arabidopsis FLOWERING LOCUS D LIKE* (*FLD-like*)*, Glyma.09G187900*, the ortholog of *Arabidopsis FT INTERACTING PROTEIN* (*FTIP*)*, Glyma.09G188000*, the ortholog of *Arabidopsis VERNALIZATION INSENSITIVE 3 LIKE* (*VIN3-like*) (**Supplementary Table S4**). Of these six genes, only three showed coding sequence differences between ZY06 and SN14 (**Supplementary Table S4**). We then screened for mutants of these three genes in a Wm82 mutant library induced by EMS and grew them in the Ningxia to evaluate flowering time. The results showed that *Glyma.09G185800* and *Glyma.09G187900* mutants exhibited significantly delayed flowering and maturity times, as well as reduced SW, consistent with the phenotype observed in the CSSL lines (**Figures 4D-I**; **Supplementary Figure S3**), indicating that *Glyma.09G185800* and *Glyma.09G187900* are the potential candidate genes. According to the phylogenetic investigation, *Glyma.09G185800* and *Glyma.09G187900* were named as *GmFLD-like1* and *GmFTIP09* (**Figure 4D**; **Supplementary Figure S4A**).We further analyzed natural variation in *GmFLD-like1* and *GmFTIP09* coding sequence using re-sequencing data from a panel of 3118 soybean accessions. The variation in *GmFLD-like1* defined 10 haplotypes. Surprisingly, none of these haplotypes matched the allele found in SN14 (**Supplementary Figures S4B, C**). In this case, we cannot use association analysis to determine whether the two haplotypes affect flowering time, so we will not focus on *GmFLD-like1* here. For *GmFTIP09*, sequence comparisons identified a total of eight haplotypes, with H1 and H4 corresponding to the SN14 and ZY06 alleles (**Supplementary Figures S5A, B**), respectively. The H1 haplotype was found in most landrace and improved cultivars, suggesting that H1 was under strict artificial selection during the early stage of modern soybean breeding (**Supplementary Figure S5C**). To further explore the functional significance of H1 and H4, we examined their association with R1 and SW. Since H4 was only found in wild soybeans, the analysis was conducted using wild soybean accessions. In three different locations, accessions with H1 haplotype flowered significantly earlier and had larger SW compared to those with the H4 haplotype, confirming the function of *GmFTIP09* on R1 and SW (**Supplementary Figures S5D-G**).

The above results suggested that *GmFTIP09* may be a candidate gene for Chr09-cluster-1. In *Arabidopsis thaliana*, *FTIP1* is involved in the florigen (*FT*) movement to the shoot apex and is associated with late flowering. Studies also showed that *FT* expression was downregulated in *ftip1* mutant (Liu et al., 2012). To determine whether *GmFTIP09* affects the expression of *GmFT2a* (the orthologs of *Arabidopsis thaliana FT*), we quantified *FT2a* transcription in SN14 and the CSSL-77. *FT2a* expression was up-regulated in SN14, indicating that the ZY06 allele (H4) repressed *FT2a* expression and resulted in the later flowering (**Figures 4J, K**).

## 4 Discussion

Cultivated soybean was domesticated from wild soybean in the Huang-Huai region (Jia et al., 2024). Compared to wild soybean accessions, cultivars exhibit significantly earlier flowering (Lu et al., 2020). In this research, we found that the allelic variations of cultivated soybeans SN14 at most QTLs associated with R1, RP, and R8 promote early flowering and maturity. This finding is consistent with previous research that early flowering time was selected during the domestication (Lu et al., 2020). However, for some QTLs, such as *qR1-6.1*/*qR8-6.1* and *qR1-16.1*/q*R8-16.1*, the allele derived from wild soybean ZY06 promote flowering time and maturity. Compared to the cultivated soybean, its wild counterparts exhibit significantly higher genetic diversity due to the not gone through the artificial selection and population bottlenecks (Hyten et al., 2006; Lam et al., 2011; Li et al., 2010). This loss of diversity in cultivated soybeans may have resulted in the loss of some valuable genes or alleles crucial environmental adaptation. Consequently, the favorable alleles identified in ZY06 serve as a good source that can be re-introduced into soybean cultivars to breeding elite soybeans. In wild soybeans, only *E1*, *E1La*, *E1Lb*, *Tof5*, and *Tof12* were reported to condition wild soybean for early flowering in high latitude (Lu et al., 2020; Dong et al., 2022, 2023; Fang et al., 2024b). Our reported QTLs, such as *qR1-6.1*/*qR8-6.1* and *qR1-16.1*/q*R8-16.1*, were not overlap with the above known genes, indicating novel genetic elements. Further fine mapping the candidate for these QTLs will expand our understanding of the molecular mechanisms underlying wild soybean’s early flowering and maturity, providing valuable genetic resources for early-flowering breeding in cultivated soybean.

Based on the position of 130 QTLs, we found 16 QTL clusters. The emergence of favorable alleles in QTL clusters explains the strong phenotypic connection between crucial traits and linkage and suggests that genes in QTL clusters may be pleiotropic or deeply interconnected (Wang et al., 2020; Zhang et al., 2020). Among these clusters, Chr05-cluster-1, Chr11-cluster-2, and Chr16-cluster-1only contained the growth period QTL. This will facilitate the ecology adaptability without thinking about the yield. The other 13 clusters controlled flowing time, maturity and grain-related traits. The Chr09-cluster-1 was the most stable one, because three QTLs regulating R1, R8 and SW in this cluster were all detected in three environments. This interval contains no previously identified genes or loci known to regulate R1, R8 and SW, suggesting that the identified locus may represent a novel gene. Within this region, the coding sequence of *GmFTIP09*, a homolog of *Arabidopsis FTIP1* (Liu et al., 2012), exhibits variations between the parental lines. In rice, *Orchidaceae*, mango, sugarcane, the homolog of *Arabidopsis FTIP1* were all reported to regulate flowering, which provides evidence for a conserved role of *FTIP1* in mediating flowering (Song et al., 2017; Wang et al., 2017; Yadav et al., 2020; Fang et al., 2024a). In soybean, association analysis in a natural population (based on haplotypes) revealed that *GmFTIP09* regulates R1, R8 and SW, implicating it as a promising candidate gene for this locus.

In *Arabidopsis*, *FTIP1* regulated flowering time, but *FTIP1* expression was not regulated by known flowering genetic pathways (Liu et al., 2012). This regulatory pattern suggests that *FTIP1* is less influenced by the environment, which is consistent with the stable results detected for the cluster under different environmental conditions. Interestingly, we found that the early-flowering haplotype of *GmFTIP09* underwent strong selection and fixation during domestication. Therefore, this locus can only be detected in a genetic population derived from the cross between cultivated and wild soybean.

*FTIP1* regulated flowering time by two ways in *Arabidopsis*, one is that *FTIP1* affecting *FT* transport through the phloem to the shoot apical meristem, the other is that *FTIP1* regulated the *FT* mRNA expression. In this research, we detected the transcriptional level between the parental haplotype and found that the SN14 haplotype relieves the inhibitory effect of *GmFTIP09* on *FT*, promoting soybean flowering. In addition, we found that *FTIP09* regulate seed weight, but the molecular mechanism remains unclear. *FT* is the member of the functional evolution of phosphatidylethanolamine binding proteins (PEBP) family, which can be divided into three subfamilies, *TERMINAL FLOWER1*(*TFL1*)-like, *FLOWERING LOCUS T*(*FT*)-like, and *MOTHER OF FT AND TFL1*(*MFT*)-like (Danilevskaya et al., 2008). Both of the *GmMFT* (Cai et al., 2023), the number of *MFT* subfamily, and *Dt1* (Li et al., 2024a), the number of *TFL1-like* subfamily, were reported to regulated seed weight in soybean. In rice, *FT-like* 9 (*FTL9*), the member of the FT protein family, regulates grain size (Ta et al., 2023). These results lead us to guess *FTIP* regulated seed weight through the *FT* in soybean. However, *FT* exhibited only minor effect on seed size, but a significantly larger surface in *Arabidopsis* (Bigas et al., 2025). Therefore, further investigation is needed to explore the molecular mechanisms of *FTIP* in regulating grain size and to clarify whether *FT* participates in the regulation of soybean grain weight.

In addition, soybean roots have nodules in which symbiotic bacteria fixed nitrogen to host plants. GmFTs protein can move from the shoots to the roots and in soybean hairy roots enhanced nodule formation (Wang et al., 2021). In *Arabidopsis, FTIP1* affecting *FT* transport through the phloem to the shoot apical meristem. Whether the *FTIP* affecting the *FT* transport to the root and whether *FTIP* regulated the symbiotic nitrogen fixation deserved further research.

## 5 Conclusion

In this study, 88 QTLs for growth period and 42 QTLs for seed-related traits were detected, and 16 clusters were defined. Focusing on the most stable QTL cluster, combining homologous alignment analysis, haplotype analysis and RT-qPCR, *GmFTIP09* was identified as the target gene associated with growth period and grain traits. The discovery of newly detected QTL loci and the mining of candidate genes provide valuable insight for breeding soybean cultivars with high yield and geographical distribution.

## Data Availability

The datasets presented in this study can be found in online repositories. The names of the repository/repositories and accession number(s) can be found in the article/**Supplementary Material**.
