# Supplementary material for: GmFTIP09 regulated flowering time and seed weight
Source: Front Plant Sci. 2025 Aug 11;16:1640116. doi: 10.3389/fpls.2025.1640116 (PMC12375491; doi:10.3389/fpls.2025.1640116)
Supplement: Supplementary file 1 [file Image1.pdf]

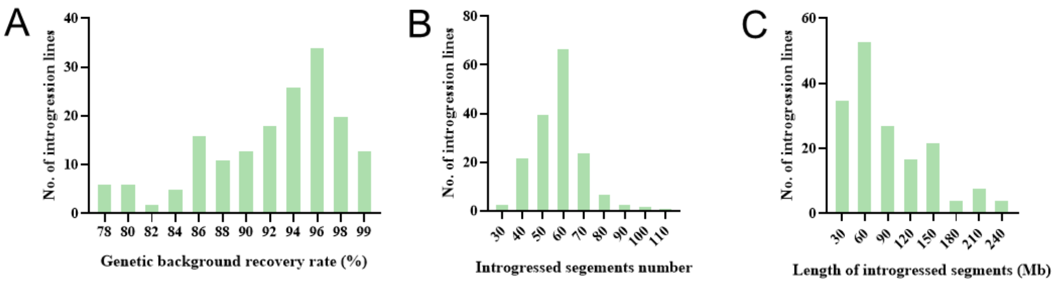

Figure S1. Genetic constitution and introgressive segments of CSSLs.

A: Genetic background recovery rate; **B:** Number of introgressed segments in the CSSLs population; **C:** Length of introgressed segments in the CSSLs population.

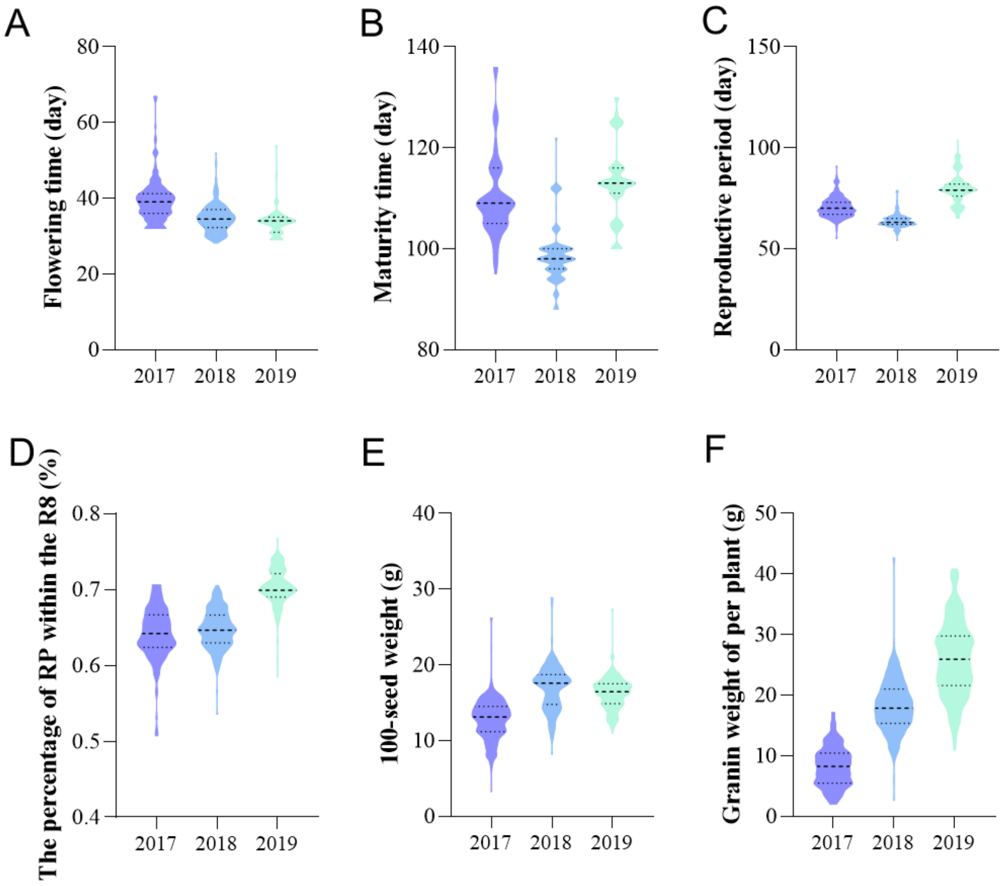

Figure S2. The phenotypic performance of CSSLs in 2017, 2018 and 2019.

A: Flowering time (R1); **B:** Maturity (R8); **C:** Reproductive period (RP); **D:** The percentage of RP within the R8 (PM); **E:** 100-seed weight (SW); **F:** Grain weight of per plant (GW).

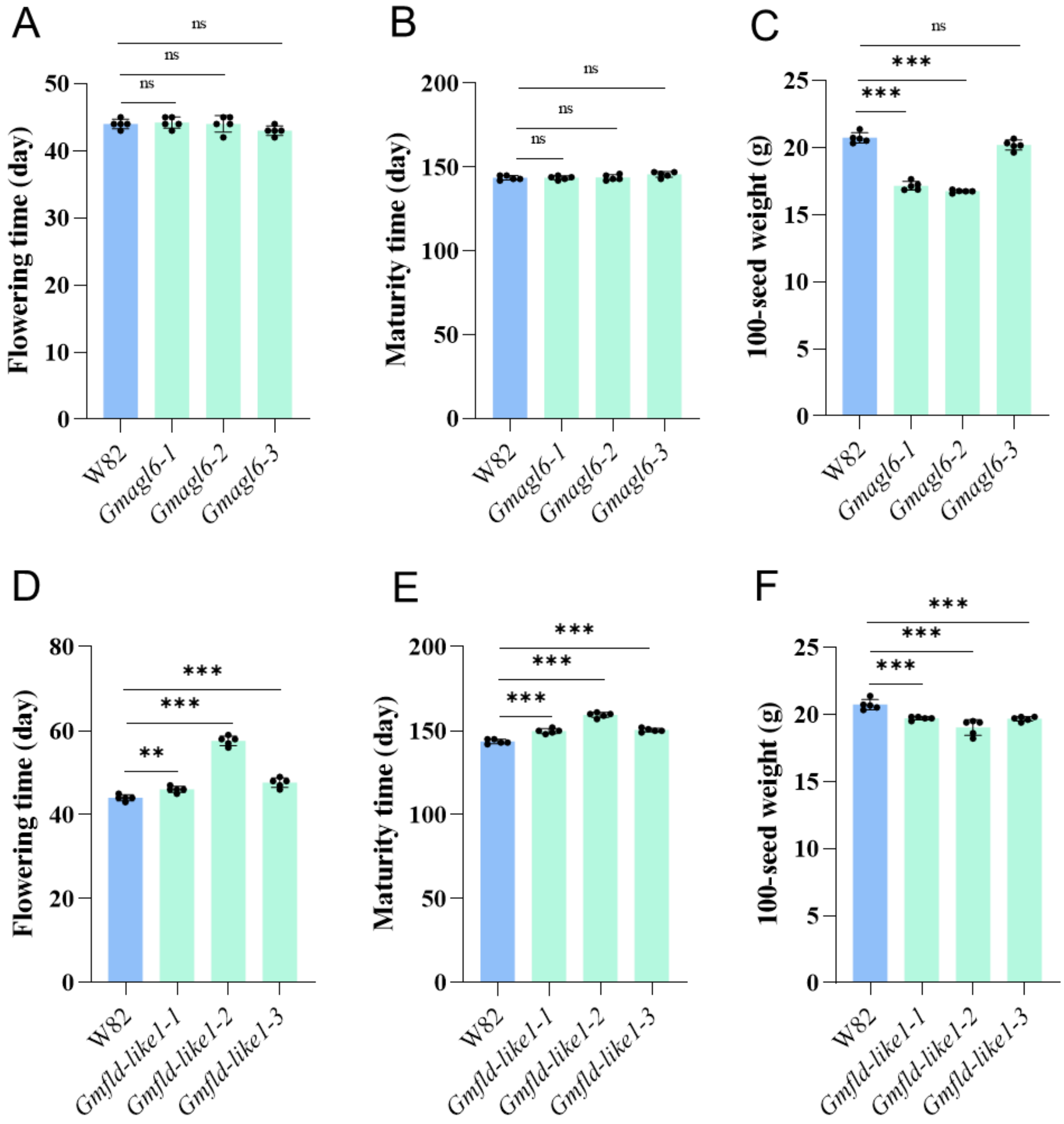

Figure S3. The phenotypic performance of *Gmagl6* and *Gmfld-like1* in W82 EMS mutant.

A-C: The phenotypic performance of *Gmagl6* mutant in Ningxia. The EMS mutant of *Gmagl6* has three different mutation types, named *Gmagl6-1*, *Gmagl6-2*, and *Gmagl6-3*. Compared with Wm82, *Gmagl6-1* harbors one non-synonymous variations at the coding sequence (CDS) positions of 3 and caused a conversion of the 1th amino acid from glycine to aspartic acid (M-I); *Gmagl6-2* harbors one non-synonymous variations at the CDS positions of 11 and caused a conversion of the 4th amino acid from glutamic acid to lysine (G-E); *Gmagl6-3*, harbors one non-synonymous variations at the CDS positions of 146 and caused a conversion of the 49th amino acid from alanine to threonine (S-F). **A:** Flowering time (R1); **B:** Maturity (R8); **C:** 100-seed weight (SW). All data are means ± SD (n=5). **D-F:** The phenotypic performance of *Gmfld-like1* mutant in Ningxia. The EMS mutant of *Gmfld-like1* has three different mutation types, named *Gmfld-like1-1*, *Gmfld-like1-2*, and *Gmfld-like1-3*. Compared with Wm82, *Gmfld-like1-1* harbors one non-synonymous variations at the coding sequence (CDS) positions of 325 and caused a conversion of the 109th amino acid from glycine to aspartic acid (D-N); *Gmfld-like1-2* harbors one non-synonymous variations at the CDS positions of 1877 and caused a conversion of the 626th amino acid from glutamic acid to lysine

(A-V); *Gmfld-like1-3*, harbors one non-synonymous variations at the CDS positions of 2113 and caused a conversion of the 705th amino acid from alanine to threonine (P-S). **D:** Flowering time (R1); **E:** Maturity (R8); **F:** 100-seed weight (SW). All data are means ± SD (n=5). The two-sided Student's t-test was performed to determine statistically significant differences in **A-F**. *, ** and *** represent significant differences at the 0.05, 0.01 and 0.001 probability levels, respectively.

A

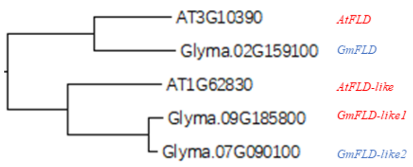

B

REF	T	C	C	T	A	A	ATTTGTTTTTGGATGAT<br>ACAAGTATTAAATTTCTT	CTGCCTGACCCGTATATGA<br>TTCTTTTGAATGATGACAT	A	T	G
ALT	A	T	G	C	T	T	A	C	G	A	A
POS	41085584	41086566	41086587	41086593	41086848	41087908	41087925	41087964	41088012	41088013	41088040
H1	T	C	C	T	A	A	ATTTGTTTTTGGATGAT<br>ACAAGTATTAAATTTCTT	CTGCCTGACCCGTATATGA<br>TTCTTTTGAATGATGACAT	A	T	G
H2	A	C	C	T	A	A	ATTTGTTTTTGGATGAT<br>ACAAGTATTAAATTTCTT	CTGCCTGACCCGTATATGA<br>TTCTTTTGAATGATGACAT	A	T	G
H3	T	C	C	T	A	T	ATTTGTTTTTGGATGAT<br>ACAAGTATTAAATTTCTT	CTGCCTGACCCGTATATGA<br>TTCTTTTGAATGATGACAT	A	T	G
H4	A	C	C	T	A	T	ATTTGTTTTTGGATGAT<br>ACAAGTATTAAATTTCTT	CTGCCTGACCCGTATATGA<br>TTCTTTTGAATGATGACAT	A	T	G
H5	T	C	C	T	A	A	A	C	G	T	G
H6	A	C	C	T	A	A	A	C	G	T	G
H7	T	C	C	T	A	T	ATTTGTTTTTGGATGAT<br>ACAAGTATTAAATTTCTT	CTGCCTGACCCGTATATGA<br>TTCTTTTGAATGATGACAT	A	A	G
H8	A	C	C	T	A	T	ATTTGTTTTTGGATGAT<br>ACAAGTATTAAATTTCTT	CTGCCTGACCCGTATATGA<br>TTCTTTTGAATGATGACAT	A	A	G
H9	T	C	C	T	A	A	ATTTGTTTTTGGATGAT<br>ACAAGTATTAAATTTCTT	CTGCCTGACCCGTATATGA<br>TTCTTTTGAATGATGACAT	A	T	A
H10	A	C	C	T	A	A	ATTTGTTTTTGGATGAT<br>ACAAGTATTAAATTTCTT	CTGCCTGACCCGTATATGA<br>TTCTTTTGAATGATGACAT	A	T	A
CDS nt# in Willianms 82	2469	422	1466	1460	1205	145	93_127del	52_88del	41	40	13
AA nt# in Willianms 82	823	141	489	487	402	49	31	18	14	14	5
AA identified in Williams 82	L	F	G	Y	M	S	Q	M	M	M	Q
AA change	F	Y	A	C	K	T	fs	fs	T	L	X

C

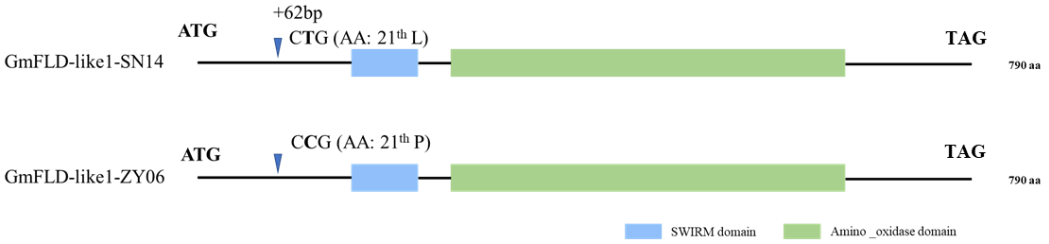

Figure S4. Analysis for the candidate gene *GmFLD-like1*.

A: Phylogenetic tree based on the amino acid sequences of FLD proteins and their homologous proteins using the neighbor-joining method. **B:** Summary of the main haplotypes for the candidate gene *GmFLD-like1*. REF represent W82, ALT represent different with W82. **C:** The amino acid sequences different of *GmFLD-like1* between two parents. The triangle symbol represents the base mutation position. The blue bars represent SWIRM domain, green bars represent Amino_oxidase domain.

A

REF	T	C	C	T	A	G	A	T	C	A	A
ALT	C	A	T	C	G	A	T	C	T	C	T
POS	41247626	41247780	41247940	41248196	41248405	41248789	41249120	41249435	41249504	41249698	41249807
H1	T	C	C	T	A	G	A	T	C	A	A
H2	T	C	C	T	A	G	A	T	C	A	T
H3	T	C	C	C	A	G	A	T	C	A	T
H4	T	C	C	T	G	G	A	T	C	A	T
H5	C	A	T	T	A	G	T	T	T	A	T
H6	C	A	T	C	A	G	T	T	T	A	T
H7	T	C	C	T	A	G	A	T	C	C	T
H8	T	C	C	T	A	G	A	C	C	C	T
CDS nt# in Willianms 82	2161	2007	1847	1591	1382	998	667	490	421	227	118
AA nt# in Willianms 82	721	669	616	531	461	333	223	164	141	76	40
AA identified in Williams 82	M	M	C	M	M	P	S	K	A	F	W
AA change	V	I	Y	V	T	L	T	E	T	C	R

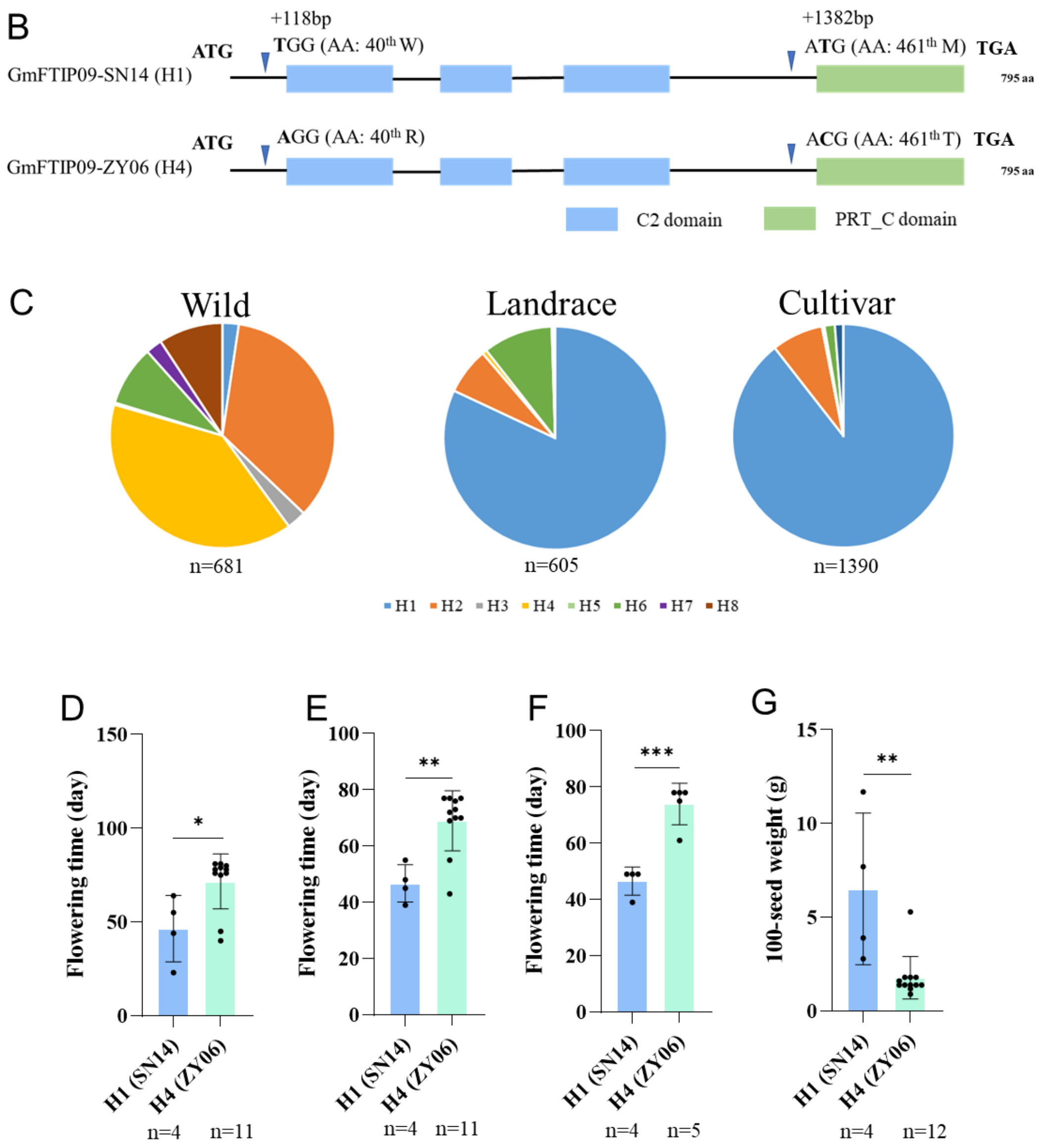

Figure S5. Summary of the main haplotypes for the candidate gene *GmFTIP09*.

A: Summary of the main haplotypes for the candidate gene *GmFTIP09*. REF represent W82 sequence, ALT represent different with W82 sequence. **B:** The amino acid sequences different of *GmFTIP09* between two parents. The triangle symbol represents the base mutation position. The blue bars represent C2 domain, green bars represent PRT_C domain. **C:** Haplotypes of *GmFTIP09* within each of the three germplasm groups. **D-F:** Flowering time

variations in wild accession possess H1 (SN14 haplotypes) and H4 (ZY06 haplotype) at *GmFTIP09* in Zhengzhou 2018 (D), Zhengzhou 2019 (E), Hefei 2018 (F). **G:** 100-seed weight variations in wild accession possess H1 (SN14 haplotypes) and H4 (ZY06 haplotype) at *GmFTIP09* in 2018 Hefei. The two-sided Student's t-test was performed to determine statistically significant differences in **D-G**. *, ** and *** represent significant differences at the 0.05, 0.01 and 0.001 probability levels, respectively.
