# Supplementary material for: GmFTIP09 regulated flowering time and seed weight
Source: Front Plant Sci. 2025 Aug 11;16:1640116. doi: 10.3389/fpls.2025.1640116 (PMC12375491; doi:10.3389/fpls.2025.1640116)
Supplement: Supplementary file 2 [file Table1.docx]

| **Table S1. RT-qPCR primer** | |
| --- | --- |
| Primer | Sequence |
| RT-GmFTIP09-F | ATGGGTGGTGATCATTGCTT |
| RT-GmFTIP09-R | CACAGTACACAGCACCATG |
| RT-GmFT2a-F | GGATTGCCAGTTGCTGCTGT |
| RT-GmFT2a-R | GAGTGTGGGAGATTGCCAAT |
|  |  |

| **Table S2. Analysis of traits phenotypic variance and heritability** | | | |
| --- | --- | --- | --- |
| Trait | Phenotypic variance | V(G)/V(P) | V(GE)/V(P) |
| R1 | 27.94 | 0.69 | 0.31 |
| R8 | 41.34 | 0.74 | 0.26 |
| RP | 26.76 | 0.38 | 0.62 |
| PM | 0.17 | 0.40 | 0.60 |
| SW | 18.93 | 0.65 | 0.35 |
| GW | 22.95 | 0.23 | 0.77 |

V(G)/V(P), variance of genetic main effects divided by phenotypic variance;

V(GE)/V(P), variance of genotype-by-environment interaction effects divided by phenotypic variance.

| **Table S3. QTL mapping for growth period and grain traits** | | | | | | | | | | |
| --- | --- | --- | --- | --- | --- | --- | --- | --- | --- | --- |
| Trait | QTL | Chromosome | Environment | Nearest marker | Position (bp) | LOD | PVE  (%) | Additive effect | Marker interval | Physical distance interval (bp) |
| R1 | *qR1-1.1* | Chr01 | 2017 | Block1 | 24901 | 6.09 | 15.20 | 3.67 | Block1-Block66 | 24901-2103472 |
|  |  |  | 2018 | Block1 | 24901 | 4.44 | 11.40 | 2.06 | Block1-Block63 | 24901-1927034 |
|  |  |  | 2019 | Block33 | 1054230 | 2.83 | 7.50 | 2.36 | Block1-Block63 | 24901-1927034 |
|  | *qR1-2.1* | Chr02 | 2018 | Block692 | 10581533 | 2.64 | 6.90 | -1.86 | Block683-Block698 | 10288831-12618932 |
|  | *qR1-3.1* | Chr03 | 2017 | Block1446 | 5805141 | 8.31 | 20.10 | 4.41 | Block1403-Block1701 | 5280717-12410844 |
|  |  |  | 2018 | Block1446 | 5805141 | 6.48 | 16.20 | 2.60 | Block1403-Block1701 | 5280717-12410844 |
|  |  |  | 2019 | Block1448 | 6027141 | 5.37 | 14.49 | 13.70 | Block1403-Block1701 | 5280717-12410844 |
|  | *qR1-3.2* | Chr03 | 2017 | Block1866 | 26733366 | 8.01 | 19.50 | 4.36 | Block1714-Block1895 | 12931270-33054917 |
|  |  |  | 2018 | Block1867 | 27566585 | 6.67 | 16.60 | 2.63 | Block1722-Block1895 | 12988232-33054917 |
|  |  |  | 2019 | Block1714 | 12931270 | 6.32 | 15.90 | 1.15 | Block1710-Block1887 | 12540058-32960034 |
|  | *qR1-3.3* | Chr03 | 2017 | Block1959 | 36409722 | 2.55 | 6.70 | 3.57 | Block1940-Block1961 | 35646249-37033892 |
|  | *qR1-4.1* | Chr04 | 2019 | Block2118 | 4116215 | 2.95 | 7.80 | 2.86 | Block2109-Block2133 | 2444540-5817945 |
|  | *qR1-4.2* | Chr04 | 2017 | Block2854 | 51171018 | 4.07 | 10.40 | 2.16 | Block2851-Block2855 | 50273860-51473445 |
|  |  |  | 2019 | Block2854 | 51171018 | 8.48 | 20.80 | 1.48 | Block2851-Block2855 | 50273860-51473445 |
|  | *qR1-5.1* | Chr05 | 2017 | Block3151 | 34859681 | 2.87 | 7.50 | -3.24 | Block3103-Block3181 | 33335930-37282803 |
|  |  |  | 2018 | Block3151 | 34859681 | 3.71 | 9.60 | -2.35 | Block3103-Block3181 | 33335930-37282803 |
|  |  |  | 2019 | Block3131 | 34196372 | 2.65 | 7.00 | -1.96 | Block3089-Block3181 | 33065545-37282803 |
|  | *qR1-6.1* | Chr06 | 2018 | Block3364 | 9351304 | 2.43 | 6.40 | -1.59 | Block3357-Block3367 | 8080039-9754620 |
|  | *qR1-6.2* | Chr06 | 2017 | Block3647 | 48917297 | 3.23 | 8.40 | 2.14 | Block3631-Block3647 | 48576391-48917297 |
|  |  |  | 2018 | Block3639 | 48766650 | 3.91 | 10.10 | 1.49 | Block3603-Block3692 | 48351919-50350663 |
|  |  |  | 2019 | Block3647 | 48917297 | 5.81 | 14.70 | 1.92 | Block3581-Block3692 | 46905031-50350663 |
|  | *qR1-7.1* | Chr07 | 2017 | Block3761 | 895883 | 6.26 | 15.60 | 5.33 | Block3730-Block3777 | 1517-1261223 |
|  |  |  | 2018 | Block3761 | 895883 | 5.69 | 14.40 | 3.21 | Block3730-Block3777 | 1517-1261223 |
|  |  |  | 2019 | Block3761 | 895883 | 3.59 | 9.40 | 2.79 | Block3730-Block3777 | 1517-1261223 |
|  | *qR1-9.1* | Chr09 | 2017 | Block4856 | 37598188 | 16.51 | 36.10 | 9.88 | Block4816-Block4882 | 28432439-44378928 |
|  |  |  | 2018 | Block4856 | 37598188 | 10.6 | 25.10 | 5.74 | Block4853-Block4870 | 37541828-43618997 |
|  |  |  | 2019 | Block4856 | 37598188 | 11.85 | 27.70 | 5.81 | Block4816-Block4859 | 28432439-41584174 |
|  | *qR1-9.2* | Chr09 | 2017 | Block4898 | 45588895 | 5.18 | 13.10 | 3.16 | Block4889-Block4961 | 44842178-49802859 |
|  |  |  | 2018 | Block4918 | 46794304 | 2.45 | 6.40 | 1.47 | Block4898-Block4933 | 45588895-47542984 |
|  |  |  | 2019 | Block4933 | 47542984 | 4.42 | 11.40 | 1.29 | Block4874-Block4933 | 43700046-47542984 |
|  | *qR1-10.1* | Chr10 | 2017 | Block4970 | 1928029 | 4.69 | 11.90 | 3.47 | Block4963-Block4970 | 1242-1928029 |
|  |  |  | 2018 | Block4968 | 1745291 | 2.34 | 6.20 | 1.61 | Block4967-Block4970 | 1675325-1928029 |
|  |  |  | 2019 | Block4973 | 2427984 | 8.52 | 20.80 | 3.38 | Block4963-Block4973 | 1242-2427984 |
|  | *qR1-10.2* | Chr10 | 2019 | Block5358 | 42829695 | 2.21 | 5.90 | 4.58 | Block5355-Block5363 | 42360768-43479261 |
|  | *qR1-10.3* | Chr10 | 2017 | Block5386 | 45666079 | 16.04 | 35.20 | 7.04 | Block5385-Block5421 | 45295261-48433614 |
|  |  |  | 2018 | Block5386 | 45666079 | 14.54 | 32.70 | 4.50 | Block5372-Block5421 | 44920705-48433614 |
|  |  |  | 2019 | Block5387 | 45811481 | 29.42 | 55.40 | 6.15 | Block5372-Block5421 | 44920705-48433614 |
|  | *qR1-11.1* | Chr11 | 2017 | Block5784 | 29946683 | 2.75 | 7.20 | 4.25 | Block5782-Block5784 | 28506663-29946683 |
|  |  |  | 2018 | Block5784 | 29946683 | 2.52 | 6.60 | 2.53 | Block5782-Block5784 | 28506663-29946683 |
|  | *qR1-11.2* | Chr11 | 2017 | Block5810 | 31478267 | 5.2 | 13.10 | 7.25 | Block5810-Block5830 | 31478267-33124982 |
|  |  |  | 2018 | Block5829 | 32773276 | 2.55 | 6.70 | 2.34 | Block5829-Block5830 | 32773276-33124982 |
|  | *qR1-12.1* | Chr12 | 2017 | Block5856 | 640400 | 6.91 | 17.10 | 4.42 | Block5840-Block5957 | 3971-6797211 |
|  |  |  | 2018 | Block5949 | 5359537 | 5.53 | 14.00 | 2.63 | Block5856-Block5957 | 640400-6797211 |
|  |  |  | 2019 | Block5884 | 2544773 | 4.65 | 12.00 | 2.65 | Block5840-Block5952 | 3971-5678851 |
|  | *qR1-14.1* | Chr14 | 2017 | Block7370 | 15499592 | 2.68 | 7.00 | 3.74 | Block7370-Block7371 | 15499592-15584998 |
|  |  |  | 2019 | Block7430 | 19121582 | 3.43 | 9.00 | 1.27 | Block7367-Block7439 | 15198507-19788050 |
|  | *qR1-14.2* | Chr14 | 2017 | Block7591 | 35093819 | 2.68 | 7.00 | 3.74 | Block7530-Block7685 | 33871908-41301126 |
|  |  |  | 2019 | Block7650 | 38956484 | 6.32 | 15.90 | 1.15 | Block7503-Block7697 | 24165997-42474547 |
|  | *qR1-14.3* | Chr14 | 2017 | Block7732 | 45387555 | 8.35 | 20.30 | 11.15 | Block7720-Block7758 | 44446580-47208538 |
|  |  |  | 2018 | Block7732 | 45387555 | 5.06 | 12.90 | 7.56 | Block7721-Block7755 | 44567696-46972878 |
|  |  |  | 2019 | Block7754 | 46863934 | 7.68 | 19.00 | 2.90 | Block7724-Block7758 | 44627234-47208538 |
|  | *qR1-16.1* | Chr16 | 2017 | Block8903 | 33722577 | 8.17 | 19.90 | -2.95 | Block8822-Block8994 | 30343054-37519056 |
|  |  |  | 2018 | Block8903 | 33722577 | 9.71 | 23.20 | -2.01 | Block8816-Block8994 | 29103156-37519056 |
|  |  |  | 2019 | Block8903 | 33722577 | 11.57 | 27.20 | -1.77 | Block8822-Block8952 | 30343054-35958634 |
|  | *qR1-17.1* | Chr17 | 2017 | Block9293 | 39746095 | 2.97 | 7.70 | 3.17 | Block9283-Block9303 | 39446371-40015920 |
|  |  |  | 2018 | Block9292 | 39720641 | 3.96 | 10.20 | 2.32 | Block9277-Block9303 | 39312995-40015920 |
|  |  |  | 2019 | Block9291 | 39668135 | 4.34 | 11.20 | 2.57 | Block9270-Block9316 | 39180390-40423683 |
|  | *qR1-19.1* | Chr19 | 2017 | Block10145 | 39295144 | 2.04 | 5.40 | 3.14 | Block10144-Block10147 | 39265471-39454523 |
|  |  |  |  |  |  |  |  |  |  |  |
| R8 | *qR8-1.1* | Chr01 | 2017 | Block1 | 24901 | 6.08 | 15.20 | 4.94 | Block1-Block66 | 24901-2103472 |
|  |  |  | 2018 | Block1 | 24901 | 4.17 | 10.80 | 2.91 | Block1-Block63 | 24901-1927034 |
|  |  |  | 2019 | Block1 | 24901 | 3.54 | 9.40 | 4.25 | Block1-Block39 | 24901-1169479 |
|  | *qR8-1.2* | Chr01 | 2017 | Block92 | 3427092 | 4.1 | 10.50 | 3.09 | Block92-Block107 | 3427092-4138183 |
|  |  |  | 2018 | Block92 | 3427092 | 3 | 7.90 | 1.57 | Block92-Block97 | 3427092-3582745 |
|  | *qR8-1.3* | Chr01 | 2018 | Block229 | 43372062 | 2.16 | 5.70 | -1.92 | Block204-Block250 | 43026103-44062662 |
|  | *qR8-2.1* | Chr02 | 2017 | Block756 | 29871886 | 2.89 | 7.50 | 10.49 | Block753-Block760 | 17602464-31959518 |
|  | *qR8-3.1* | Chr03 | 2017 | Block1448 | 6027141 | 5.85 | 14.70 | 5.11 | Block1446-Block1701 | 5805141-12410844 |
|  |  |  | 2018 | Block1452 | 6386350 | 4.85 | 12.40 | 3.31 | Block1446-Block1701 | 5805141-12410844 |
|  |  |  | 2019 | Block1448 | 6027141 | 5.94 | 15.20 | 5.86 | Block1446-Block1701 | 5805141-12410844 |
|  | *qR8-3.2* | Chr03 | 2017 | Block1867 | 27566585 | 7.02 | 17.30 | 5.43 | Block1714-Block1895 | 12931270-33054917 |
|  |  |  | 2018 | Block1857 | 25304404 | 4.89 | 12.50 | 3.39 | Block1725-Block1895 | 13179139-33054917 |
|  |  |  | 2019 | Block1876 | 28124415 | 6.01 | 15.40 | 5.59 | Block1725-Block1919 | 13179139-34582995 |
|  | *qR8-3.3* | Chr03 | 2017 | Block1961 | 37033892 | 5.48 | 13.80 | 5.22 | Block1938-Block1980 | 35391537-38110602 |
|  |  |  | 2018 | Block1961 | 37033892 | 4.38 | 11.30 | 3.53 | Block1959-Block1980 | 36409722-38110602 |
|  |  |  | 2019 | Block1961 | 37033892 | 4.11 | 10.80 | 6.33 | Block1940-Block1980 | 35646249-38110602 |
|  | *qR8-4.1* | Chr04 | 2019 | Block2526 | 20269631 | 2.1 | 5.60 | -2.86 | Block2526-Block2527 | 20269631-21552318 |
|  | *qR8-4.2* | Chr04 | 2019 | Block2594 | 31958157 | 2.1 | 5.60 | -2.86 | Block2593-Block2595 | 31029349-32600033 |
|  | *qR8-4.3* | Chr04 | 2017 | Block2854 | 51171018 | 5.78 | 14.50 | 3.50 | Block2851-Block2874 | 50273860-51627705 |
|  |  |  | 2018 | Block2853 | 51008960 | 2.64 | 7.00 | 2.12 | Block2853-Block2854 | 51008960-51171018 |
|  | *qR8-5.1* | Chr05 | 2018 | Block3126 | 34038370 | 2.67 | 7.10 | -2.70 | Block3103-Block3176 | 33335930-36692594 |
|  | *qR8-6.1* | Chr06 | 2017 | Block3364 | 9351304 | 3.47 | 9.00 | -3.26 | Block3318-Block3367 | 6249666-9754620 |
|  |  |  | 2018 | Block3364 | 9351304 | 5.12 | 13.10 | -3.26 | Block3306-Block3370 | 5613196-10225296 |
|  |  |  | 2019 | Block3365 | 9512030 | 4.47 | 11.70 | -4.33 | Block3330-Block3370 | 7212177-10225296 |
|  | *qR8-7.1* | Chr07 | 2017 | Block4023 | 38661809 | 2.46 | 6.40 | -4.16 | Block3988-Block4039 | 36150737-39783766 |
|  |  |  | 2018 | Block4023 | 38661809 | 3.93 | 10.20 | -3.79 | Block3987-Block4039 | 36109308-39783766 |
|  |  |  | 2019 | Block4023 | 38661809 | 3.59 | 9.50 | -5.06 | Block3992-Block4039 | 36519641-39783766 |
|  | *qR8-9.1* | Chr09 | 2017 | Block4858 | 40609085 | 10.76 | 25.30 | 10.44 | Block4816-Block4865 | 28432439-42019955 |
|  |  |  | 2018 | Block4858 | 40609085 | 6.26 | 15.80 | 6.01 | Block4853-Block4882 | 37541828-44378928 |
|  |  |  | 2019 | Block4858 | 40609085 | 5.26 | 13.60 | 8.55 | Block4856-Block4882 | 37598188-44378928 |
|  | *qR8-9.2* | Chr09 | 2017 | Block4898 | 45588895 | 6.81 | 16.80 | 4.88 | Block4889-Block4933 | 44842178-47542984 |
|  |  |  | 2018 | Block4933 | 47542984 | 6.22 | 15.70 | 1.81 | Block4892-Block4933 | 44881908-47542984 |
|  |  |  | 2019 | Block4933 | 47542984 | 5.69 | 14.60 | 3.35 | Block4892-Block4933 | 44881908-47542984 |
|  | *qR8-10.1* | Chr10 | 2017 | Block5386 | 45666079 | 11.28 | 26.30 | 8.22 | Block5355-Block5407 | 42360768-46838038 |
|  |  |  | 2018 | Block5386 | 45666079 | 9.94 | 23.80 | 5.55 | Block5355-Block5407 | 42360768-46838038 |
|  |  |  | 2019 | Block5386 | 45666079 | 10.52 | 25.30 | 9.16 | Block5355-Block5421 | 42360768-48433614 |
|  | *qR8-11.1* | Chr11 | 2017 | Block5717 | 11089394 | 4.34 | 11.10 | 6.13 | Block5708-Block5728 | 10258092-11589308 |
|  |  |  | 2018 | Block5719 | 11121477 | 5.77 | 14.60 | 4.78 | Block5713-Block5728 | 10682661-11589308 |
|  |  |  | 2019 | Block5719 | 11121477 | 2.53 | 6.80 | 4.98 | Block5717-Block5728 | 11089394-11589308 |
|  | *qR8-11.2* | Chr11 | 2017 | Block5761 | 24385343 | 13.34 | 30.30 | 8.87 | Block5748-Block5778 | 14414197-27935742 |
|  |  |  | 2018 | Block5761 | 24385343 | 10.72 | 25.50 | 5.73 | Block5747-Block5784 | 13970351-29946683 |
|  |  |  | 2019 | Block5761 | 24385343 | 6.51 | 16.50 | 7.08 | Block5749-Block5778 | 14889439-27935742 |
|  | *qR8-11.3* | Chr11 | 2017 | Block5810 | 31478267 | 3.43 | 8.90 | 8.07 | Block5810-Block5830 | 31478267-33124982 |
|  |  |  | 2018 | Block5810 | 31478267 | 3.76 | 9.80 | 5.77 | Block5810-Block5830 | 31478267-33124982 |
|  |  |  | 2019 | Block5810 | 31478267 | 3.14 | 8.30 | 7.94 | Block5810-Block5830 | 31478267-33124982 |
|  | *qR8-12.1* | Chr12 | 2017 | Block5949 | 5359537 | 13.7 | 31.00 | 8.06 | Block5856-Block5965 | 640400-7915643 |
|  |  |  | 2018 | Block5949 | 5359537 | 7.71 | 19.00 | 4.44 | Block5856-Block5967 | 640400-7950034 |
|  |  |  | 2019 | Block5949 | 5359537 | 7.87 | 19.60 | 7.03 | Block5856-Block5965 | 640400-7915643 |
|  | *qR8-14.1* | Chr14 | 2017 | Block7754 | 46863934 | 5.87 | 14.70 | 8.13 | Block7720-Block7758 | 44446580-47208538 |
|  |  |  | 2018 | Block7732 | 45387555 | 4.81 | 12.30 | 11.75 | Block7724-Block7755 | 44627234-46972878 |
|  |  |  | 2019 | Block7724 | 44627234 | 3.36 | 8.90 | 5.56 | Block7721-Block7735 | 44567696-45474817 |
|  | *qR8-15.1* | Chr15 | 2018 | Block8036 | 22963449 | 2.84 | 7.50 | -2.62 | Block7927-Block8573 | 15413527-48877249 |
|  |  |  | 2019 | Block8053 | 23350349 | 2.37 | 6.40 | -3.70 | Block8034-Block8565 | 22551085-44632686 |
|  | *qR8-16.1* | Chr16 | 2017 | Block8903 | 33722577 | 5.05 | 12.80 | -2.29 | Block8898-Block8905 | 33654043-33766376 |
|  |  |  | 2018 | Block8903 | 33722577 | 4.86 | 12.50 | -2.13 | Block8861-Block8912 | 32145650-33981127 |
|  |  |  |  |  |  |  |  |  |  |  |
| RP | *qRP-1.1* | Chr01 | 2018 | Block246 | 43955394 | 2.87 | 7.60 | -1.54 | Block204-Block254 | 43026103-45425695 |
|  |  |  | 2019 | Block246 | 43955394 | 3.66 | 9.70 | -3.31 | Block204-Block253 | 43026103-44923213 |
|  | *qRP-2.1* | Chr02 | 2018 | Block886 | 46147372 | 3.26 | 8.50 | 1.54 | Block886-Block930 | 46147372-47873237 |
|  | *qRP-4.1* | Chr04 | 2018 | Block2224 | 10297797 | 2.92 | 7.70 | -1.78 | Block2208-Block2260 | 9931999-11217454 |
|  | *qRP-4.2* | Chr04 | 2018 | Block2649 | 41052745 | 3.45 | 9.00 | -1.52 | Block2301-Block2719 | 12195118-46014951 |
|  | *qRP-5.1* | Chr05 | 2018 | Block3257 | 41616849 | 2.77 | 7.30 | 0.49 | Block3214-Block3258 | 40172338-41738055 |
|  | *qRP-6.1* | Chr06 | 2017 | Block3364 | 9351304 | 4.04 | 10.40 | -1.52 | Block3306-Block3370 | 5613196-10225296 |
|  |  |  | 2018 | Block3363 | 8527038 | 3.37 | 8.80 | -1.64 | Block3306-Block3370 | 5613196-10225296 |
|  |  |  | 2019 | Block3365 | 951203 | 3.93 | 10.40 | -3.28 | Block3306-Block3370 | 5613196-10225296 |
|  | *qRP-7.1* | Chr07 | 2018 | Block4028 | 38964623 | 4.43 | 11.40 | -2.90 | Block3988-Block4039 | 36150737-39783766 |
|  |  |  | 2019 | Block4028 | 38964623 | 2.65 | 7.10 | -4.31 | Block3992-Block4036 | 36519641-39760852 |
|  | *qRP-9.1* | Chr09 | 2018 | Block4898 | 45588895 | 2.82 | 7.40 | 1.49 | Block4897-Block4921 | 45224216-46985214 |
|  |  |  | 2019 | Block4898 | 45588895 | 3.64 | 9.70 | 3.19 | Block4896-Block4933 | 45180567-47542984 |
|  | *qRP-10.1* | Chr10 | 2017 | Block5372 | 44920705 | 3.52 | 9.10 | 5.94 | Block5371-Block5372 | 43911358-44920705 |
|  | *qRP-11.1* | Chr11 | 2017 | Block5772 | 26206043 | 3.54 | 9.10 | 3.64 | Block5749-Block5778 | 14889439-27935742 |
|  |  |  | 2018 | Block5771 | 2584615 | 5.08 | 13.00 | 2.99 | Block5747-Block5778 | 13970351-27935742 |
|  |  |  | 2019 | Block5771 | 2584615 | 4.23 | 11.10 | 4.17 | Block5749-Block5778 | 14889439-27935742 |
|  | *qRP-11.2* | Chr11 | 2019 | Block5810 | 31478267 | 4.06 | 10.70 | 7.70 | Block5810-Block5830 | 31478267-33124982 |
|  | *qRP-12.1* | Chr12 | 2019 | Block5885 | 2764641 | 3.2 | 8.50 | 4.12 | Block5856-Block5889 | 640400-2884222 |
|  | *qRP-12.2* | Chr12 | 2017 | Block5949 | 5069087 | 7.36 | 18.00 | 3.87 | Block5906-Block5965 | 3245880-7915643 |
|  |  |  | 2018 | Block5959 | 6892695 | 4.93 | 12.60 | 2.63 | Block5933-Block5969 | 4858602-8023783 |
|  |  |  | 2019 | Block5949 | 5359537 | 6.31 | 16.10 | 5.31 | Block5906-Block5965 | 3245880-7915643 |
|  | *qRP-12.3* | Chr12 | 2018 | Block6008 | 20504796 | 2.12 | 5.70 | 1.42 | Block5998-Block6009 | 15613505-20750425 |
|  | *qRP-15.1* | Chr15 | 2017 | Block7828 | 2163645 | 3.01 | 7.80 | -2.52 | Block7790-Block7841 | 111032-3051114 |
|  |  |  | 2018 | Block7799 | 1096295 | 2.43 | 6.40 | -1.80 | Block7790-Block7819 | 111032-1826776 |
|  |  |  | 2019 | Block7797 | 858670 | 2.97 | 8.00 | -3.91 | Block7790-Block7805 | 111032-1298121 |
|  | *qRP-16.1* | Chr16 | 2018 | Block8637 | 353794 | 3.5 | 9.10 | 2.46 | Block8631-Block8658 | 106040-1464228 |
|  | *qRP-20.1* | Chr20 | 2018 | Block10659 | 29747296 | 3.23 | 8.50 | -1.69 | Block10532-Block10702 | 9044460-33571708 |
|  | *qRP-20.2* | Chr20 | 2017 | Block10775 | 36614269 | 6.94 | 17.10 | 8.14 | Block10769-Block10821 | 36433979-39526559 |
|  |  |  | 2018 | Block10775 | 36614269 | 9.12 | 22.10 | 6.50 | Block10753-Block10821 | 35870321-39526559 |
|  |  |  | 2019 | Block10776 | 36908869 | 3.06 | 8.20 | 7.84 | Block10770-Block10787 | 36490383-37413634 |
|  |  |  |  |  |  |  |  |  |  |  |
| PM | *qPM-1.1* | Chr01 | 2019 | Block246 | 43955394 | 4.2 | 11.10 | -0.01 | Block204-Block253 | 43026103-44923213 |
|  | *qPM-2.1* | Chr02 | 2018 | Block692 | 10581533 | 3.92 | 10.20 | 0.02 | Block680-Block698 | 10132887-12618932 |
|  | *qPM-2.2* | Chr02 | 2018 | Block945 | 48164277 | 4.28 | 11.10 | 0.01 | Block886-Block946 | 46147372-48194968 |
|  | *qPM-3.1* | Chr03 | 2017 | Block1587 | 10956736 | 5.05 | 12.80 | -0.02 | Block1446-Block1701 | 5805141-12410844 |
|  |  |  | 2018 | Block1449 | 6109954 | 4.05 | 10.50 | -0.01 | Block1446-Block1701 | 5805141-12410844 |
|  | *qPM-3.2* | Chr03 | 2017 | Block1726 | 13220631 | 4.2 | 10.70 | -0.02 | Block1722-Block1887 | 12988232-32960034 |
|  |  |  | 2018 | Block1877 | 28215055 | 4.21 | 10.90 | -0.01 | Block1722-Block1887 | 12988232-32960034 |
|  | *qPM-4.1* | Chr04 | 2019 | Block2113 | 3003499 | 3.89 | 10.30 | -0.02 | Block2104-Block2133 | 1678-5817945 |
|  | *qPM-5.1* | Chr05 | 2017 | Block3181 | 37282803 | 3.12 | 8.10 | 0.02 | Block3133-Block3181 | 34312139-37282803 |
|  |  |  | 2018 | Block3181 | 37282803 | 4.25 | 11.00 | 0.02 | Block3116-Block3181 | 33754807-37282803 |
|  | *qPM-6.1* | Chr06 | 2019 | Block3283 | 3531416 | 3.84 | 10.20 | -0.02 | Block3270-Block3317 | 1461351-6004701 |
|  | *qPM-6.2* | Chr06 | 2017 | Block3647 | 48917297 | 3.88 | 10.00 | -0.01 | Block3581-Block3647 | 46905031-48917297 |
|  |  |  | 2018 | Block3581 | 46905031 | 2.89 | 7.60 | -0.01 | Block3581-Block3582 | 46905031-48008828 |
|  |  |  | 2019 | Block3647 | 48917297 | 4.21 | 11.10 | -0.01 | Block3570-Block3692 | 45300917-50350663 |
|  | *qPM-7.1* | Chr07 | 2017 | Block3761 | 895883 | 3.77 | 9.70 | -0.02 | Block3730-Block3777 | 1517-1261223 |
|  |  |  | 2018 | Block3761 | 895883 | 3.46 | 9.10 | -0.02 | Block3730-Block3777 | 1517-1261223 |
|  | *qPM-10.1* | Chr10 | 2017 | Block4969 | 1807749 | 5.77 | 14.50 | -0.02 | Block4963-Block4973 | 1242-2427984 |
|  |  |  | 2018 | Block4970 | 1928029 | 2.55 | 6.70 | -0.01 | Block4967-Block4970 | 1675325-1928029 |
|  |  |  | 2019 | Block4973 | 2427984 | 7.29 | 18.40 | -0.02 | Block4963-Block4978 | 1242-2575486 |
|  | *qPM-10.2* | Chr10 | 2017 | Block5387 | 45811481 | 9.25 | 22.20 | -0.03 | Block5385-Block5414 | 45295261-47081938 |
|  |  |  | 2018 | Block5386 | 45666079 | 7.95 | 19.60 | -0.02 | Block5385-Block5409 | 45295261-46985456 |
|  |  |  | 2019 | Block5401 | 46687820 | 7.34 | 18.50 | -0.02 | Block5379-Block5421 | 45137827-48433614 |
|  | *qPM-11.1* | Chr11 | 2017 | Block5810 | 31478267 | 3.49 | 9.00 | -0.04 | Block5810-Block5830 | 31478267-33124982 |
|  | *qPM-12.1* | Chr12 | 2017 | Block5868 | 1607197 | 3.36 | 8.70 | -0.02 | Block5840-Block5889 | 3971-2884222 |
|  | *qPM-12.2* | Chr12 | 2018 | Block6008 | 20504796 | 2.46 | 6.50 | 0.01 | Block5998-Block6009 | 15613505-20750425 |
|  | *qPM-14.1* | Chr14 | 2017 | Block7724 | 44627234 | 4.86 | 12.30 | -0.03 | Block7720-Block7738 | 44446580-45711150 |
|  |  |  | 2018 | Block7721 | 44567696 | 2.68 | 7.10 | -0.01 | Block7721-Block7735 | 44567696-45474817 |
|  | *qPM-15.1* | Chr15 | 2019 | Block7797 | 858670 | 5.91 | 15.20 | -0.02 | Block7790-Block7841 | 111032-3051114 |
|  | *qPM-16.1* | Chr16 | 2017 | Block8903 | 33722577 | 7.05 | 17.40 | 0.02 | Block8822-Block8994 | 30343054-37519056 |
|  |  |  | 2018 | Block8826 | 30779317 | 8.61 | 21.00 | 0.02 | Block8817-Block8994 | 29256423-37519056 |
|  |  |  | 2019 | Block8898 | 33654043 | 7.57 | 19.00 | 0.01 | Block8822-Block8979 | 30343054-36947451 |
|  | *qPM-18.1* | Chr18 | 2017 | Block9400 | 3589508 | 6.45 | 16.00 | -0.03 | Block9364-Block9416 | 1366531-4452494 |
|  |  |  | 2018 | Block9367 | 1481947 | 2.97 | 7.80 | -0.01 | Block9364-Block9384 | 1366531-2126308 |
|  | *qPM-18.2* | Chr18 | 2017 | Block9502 | 9103586 | 3.25 | 8.40 | -0.02 | Block9430-Block9613 | 5195993-47993618 |
|  |  |  | 2018 | Block9603 | 44744346 | 2.76 | 7.30 | -0.01 | Block9432-Block9610 | 5246609-47582152 |
|  |  |  | 2019 | Block9452 | 6984904 | 2.56 | 6.90 | -0.01 | Block9447-Block9453 | 6950035-7008929 |
|  | *qPM-20.1* | Chr20 | 2018 | Block10704 | 33725575 | 4.71 | 12.30 | -0.02 | Block10533-Block10706 | 9080652-34173030 |
|  | *qPM-20.2* | Chr20 | 2018 | Block10925 | 47277820 | 6.45 | 16.50 | -0.02 | Block10880-Block10929 | 44068746-47315188 |
|  |  |  |  |  |  |  |  |  |  |  |
| SW | *qSW-1.1* | Chr01 | 2017 | Block81 | 2747498 | 6.68 | 16.70 | -1.67 | Block39-Block185 | 1169479-27204683 |
|  |  |  | 2018 | Block75 | 2363868 | 7.68 | 18.90 | -1.76 | Block33-Block185 | 105423-27204683 |
|  |  |  | 2019 | Block75 | 2363868 | 4.29 | 11.70 | -1.07 | Block73-Block184 | 2215823-26792496 |
|  | *qSW-2.1* | Chr02 | 2017 | Block414 | 3734209 | 4.16 | 10.70 | -1.42 | Block355-Block537 | 915471-6518165 |
|  |  |  | 2018 | Block414 | 3734209 | 5.09 | 12.90 | -1.57 | Block355-Block537 | 915471-6518165 |
|  |  |  | 2019 | Block482 | 4891489 | 7.77 | 20.10 | -1.34 | Block335-Block537 | 10141-6518165 |
|  | *qSW-2.2* | Chr02 | 2018 | Block945 | 48164277 | 3.81 | 9.90 | -1.19 | Block886-Block946 | 46147372-48194968 |
|  | *qSW-3.1* | Chr03 | 2017 | Block1403 | 5280717 | 4.99 | 12.70 | -0.56 | Block1403-Block1701 | 5280717-12410844 |
|  |  |  | 2018 | Block1403 | 5280717 | 5.76 | 14.50 | -0.43 | Block1362-Block1476 | 5040149-7689450 |
|  | *qSW-3.2* | Chr03 | 2017 | Block1745 | 14666163 | 2.77 | 7.30 | -1.17 | Block1722-Block1837 | 12988232-19367502 |
|  | *qSW-3.3* | Chr03 | 2017 | Block1845 | 22304411 | 2.79 | 7.30 | -1.26 | Block1839-Block1887 | 19496721-32960034 |
|  |  |  | 2018 | Block1866 | 26733366 | 2.46 | 6.50 | -1.13 | Block1845-Block1874 | 22304411-27897270 |
|  | *qSW-5.1* | Chr05 | 2018 | Block3192 | 39271251 | 3.63 | 9.40 | -1.09 | Block3185-Block3194 | 38620871-39674568 |
|  |  |  | 2019 | Block3185 | 38620871 | 3.11 | 9.30 | -0.89 | Block3185-Block3194 | 38620871-39674568 |
|  | *qSW-6.1* | Chr06 | 2018 | Block3348 | 7969230 | 3.63 | 9.40 | -1.30 | Block3306-Block3367 | 5613196-9754620 |
|  |  |  | 2019 | Block3337 | 7716203 | 4.29 | 11.70 | -1.02 | Block3306-Block3370 | 5613196-10225296 |
|  | *qSW-6.2* | Chr06 | 2017 | Block3387 | 12075274 | 2.65 | 7.00 | -2.22 | Block3379-Block3437 | 11287006-14750656 |
|  | *qSW-6.3* | Chr06 | 2017 | Block3525 | 19020896 | 4.41 | 11.30 | -1.49 | Block3483-Block3532 | 16858355-25108149 |
|  |  |  | 2018 | Block3532 | 25108149 | 4.48 | 11.50 | -1.60 | Block3483-Block3532 | 16858355-25108149 |
|  |  |  | 2019 | Block3519 | 18261709 | 4.2 | 11.50 | -1.16 | Block3483-Block3532 | 16858355-25108149 |
|  | *qSW-7.1* | Chr07 | 2017 | Block3915 | 16165141 | 2.4 | 6.30 | -1.60 | Block3899-Block3923 | 15467854-16585705 |
|  |  |  | 2018 | Block3915 | 16165141 | 4.49 | 11.50 | -2.15 | Block3887-Block3931 | 12880535-17211642 |
|  |  |  | 2019 | Block3915 | 16165141 | 3.78 | 10.40 | -1.44 | Block3893-Block3931 | 14164853-17211642 |
|  | *qSW-8.1* | Chr08 | 2017 | Block4355 | 14417132 | 2.56 | 6.70 | -1.74 | Block4353-Block4362 | 13692389-14519320 |
|  |  |  | 2018 | Block4353 | 13692389 | 4.25 | 10.90 | -2.16 | Block4351-Block4362 | 12074568-14519320 |
|  |  |  | 2019 | Block4353 | 13692389 | 4.71 | 12.70 | -1.67 | Block4351-Block4362 | 12074568-14519320 |
|  | *qSW-9.1* | Chr09 | 2017 | Block4818 | 28759216 | 5.94 | 14.90 | -2.24 | Block4799-Block4859 | 1397976-41584174 |
|  |  |  | 2018 | Block4817 | 28691059 | 8.19 | 20.00 | -2.47 | Block4799-Block4859 | 1397976-41584174 |
|  |  |  | 2019 | Block4809 | 13514382 | 6.46 | 17.10 | -1.95 | Block4799-Block4853 | 1397976-37541828 |
|  | *qSW-9.2* | Chr09 | 2018 | Block4898 | 45588895 | 2.32 | 6.10 | -1.00 | Block4895-Block4913 | 44948744-46484406 |
|  | *qSW-10.1* | Chr10 | 2018 | Block4970 | 1928029 | 8.44 | 20.60 | -1.60 | Block4963-Block4976 | 1242-2490861 |
|  | *qSW-10.2* | Chr10 | 2017 | Block5396 | 46600087 | 3.24 | 8.40 | -1.60 | Block5385-Block5403 | 45295261-46747637 |
|  |  |  | 2018 | Block5396 | 46600087 | 5.75 | 14.50 | -2.10 | Block5385-Block5414 | 45295261-47081938 |
|  | *qSW-11.1* | Chr11 | 2017 | Block5570 | 7317597 | 5.92 | 14.90 | -1.30 | Block5506-Block5605 | 3602901-8227137 |
|  |  |  | 2018 | Block5570 | 7317597 | 6.84 | 17.00 | -1.47 | Block5473-Block5636 | 1696383-8701140 |
|  |  |  | 2019 | Block5573 | 7428499 | 10.23 | 25.60 | -1.29 | Block5473-Block5605 | 1696383-8227137 |
|  | *qSW-11.2* | Chr11 | 2017 | Block5761 | 24385343 | 4.64 | 11.90 | -1.99 | Block5708-Block5778 | 10258092-27935742 |
|  | *qSW-12.1* | Chr12 | 2017 | Block5952 | 5678851 | 3.08 | 8.00 | -1.52 | Block5906-Block5952 | 3245880-5678851 |
|  |  |  | 2018 | Block5949 | 5359537 | 2.91 | 7.60 | -1.36 | Block5910-Block5952 | 3949133-5678851 |
|  | *qSW-12.2* | Chr12 | 2017 | Block6024 | 33252043 | 7.15 | 17.70 | -1.06 | Block5959-Block6096 | 6892695-39001177 |
|  |  |  | 2018 | Block6024 | 33252043 | 8.06 | 19.70 | -1.51 | Block5967-Block6096 | 7950034-39001177 |
|  |  |  | 2019 | Block6024 | 33252043 | 11.98 | 29.30 | -1.16 | Block5969-Block6096 | 8023783-39001177 |
|  | *qSW-13.1* | Chr13 | 2017 | Block6179 | 842160 | 5.83 | 14.70 | -1.72 | Block6173-Block6369 | 568509-4589041 |
|  |  |  | 2018 | Block6179 | 842160 | 5.68 | 14.30 | -1.62 | Block6173-Block6326 | 568509-2502771 |
|  |  |  | 2019 | Block6179 | 842160 | 5.04 | 13.60 | -1.17 | Block6173-Block6326 | 568509-2502771 |
|  | *qSW-13.2* | Chr13 | 2017 | Block6436 | 10189667 | 5.76 | 14.50 | -1.80 | Block6434-Block6538 | 10158767-26382434 |
|  |  |  | 2018 | Block6436 | 10189667 | 6.57 | 16.40 | -1.85 | Block6434-Block6538 | 10158767-26382434 |
|  |  |  | 2019 | Block6490 | 22951383 | 6.41 | 16.90 | -1.19 | Block6434-Block6538 | 10158767-26382434 |
|  | *qSW-14.1* | Chr14 | 2018 | Block7720 | 44446580 | 4.69 | 12.00 | -3.12 | Block7720-Block7738 | 44446580-45711150 |
|  | *qSW-15.1* | Chr15 | 2018 | Block7799 | 1096295 | 3.19 | 8.30 | -1.51 | Block7790-Block7830 | 111032-2618231 |
|  |  |  | 2019 | Block7799 | 1096295 | 2.64 | 7.40 | -1.02 | Block7790-Block7829 | 111032-2413555 |
|  | *qSW-16.1* | Chr16 | 2017 | Block8650 | 953069 | 6.79 | 16.90 | -2.32 | Block8631-Block8682 | 10604-2440896 |
|  |  |  | 2018 | Block8637 | 353794 | 5.26 | 13.40 | -2.31 | Block8631-Block8658 | 10604-1464228 |
|  |  |  | 2019 | Block8637 | 353794 | 4.86 | 13.10 | -1.62 | Block8631-Block8658 | 10604-1464228 |
|  | *qSW-16.2* | Chr16 | 2018 | Block8721 | 3092856 | 2.13 | 5.60 | 1.19 | Block8717-Block8722 | 3022600-3157060 |
|  |  |  | 2019 | Block8731 | 3798615 | 2.83 | 7.90 | 1.33 | Block8717-Block8741 | 3022600-5584890 |
|  | *qSW-16.3* | Chr16 | 2017 | Block8782 | 24775423 | 5.86 | 14.80 | -0.63 | Block8781-Block8817 | 24653156-29256423 |
|  |  |  | 2018 | Block8782 | 24775423 | 5.6 | 14.20 | -0.95 | Block8781-Block8817 | 24653156-29256423 |
|  |  |  | 2019 | Block8814 | 28917998 | 8.73 | 22.30 | -0.83 | Block8781-Block8817 | 24653156-29256423 |
|  | *qSW-17.1* | Chr17 | 2017 | Block9075 | 7309000 | 4.34 | 11.10 | -1.39 | Block9016-Block9091 | 3091788-8711541 |
|  |  |  | 2018 | Block9070 | 6595864 | 5.53 | 14.00 | -1.45 | Block9016-Block9094 | 3091788-9084498 |
|  |  |  | 2019 | Block9087 | 8392145 | 7.8 | 20.20 | -1.32 | Block9016-Block9094 | 3091788-9084498 |
|  | *qSW-20.1* | Chr20 | 2019 | Block10880 | 44068746 | 4.32 | 11.80 | -0.81 | Block10858-Block10882 | 41240171-44222749 |
|  |  |  |  |  |  |  |  |  |  |  |
| GW | *qGW-1.1* | Chr01 | 2018 | Block230 | 43407966 | 2.27 | 6.00 | -1.91 | Block204-Block252 | 43026103-44655010 |
|  | *qGW-5.1* | Chr05 | 2018 | Block3187 | 38681318 | 3.45 | 9.00 | -1.91 | Block3185-Block3201 | 38620871-39907103 |
|  | *qGW-6.1* | Chr06 | 2018 | Block3322 | 6513663 | 2.53 | 6.70 | -1.90 | Block3315-Block3329 | 5956156-6985266 |
|  | *qGW-6.2* | Chr06 | 2018 | Block3340 | 7832840 | 2.66 | 7.00 | -1.91 | Block3335-Block3367 | 7620608-9754620 |
|  | *qGW-6.3* | Chr06 | 2018 | Block3582 | 48008828 | 3.4 | 8.90 | -2.10 | Block3581-Block3583 | 46905031-48118192 |
|  | *qGW-10.1* | Chr10 | 2018 | Block4963 | 1242 | 3.29 | 8.60 | -2.31 | Block4963-Block4970 | 1242-1928029 |
|  | *qGW-14.1* | Chr14 | 2018 | Block7519 | 33247938 | 2.78 | 7.30 | -1.45 | Block7503-Block7530 | 24165997-33871908 |
|  | *qGW-14.2* | Chr14 | 2018 | Block7650 | 38956484 | 3.47 | 9.00 | -1.55 | Block7576-Block7655 | 34755312-39142194 |
|  | *qGW-14.3* | Chr14 | 2018 | Block7697 | 42474547 | 2.99 | 7.80 | -1.58 | Block7686-Block7697 | 41357390-42474547 |
|  | *qGW-14.4* | Chr14 | 2017 | Block7720 | 44446580 | 3.36 | 8.80 | -3.10 | Block7720-Block7738 | 44446580-45711150 |
|  |  |  | 2018 | Block7720 | 44446580 | 3.53 | 9.20 | -4.15 | Block7720-Block7738 | 44446580-45711150 |
|  | *qGW-15.1* | Chr15 | 2019 | Block7821 | 1926425 | 3.08 | 8.50 | -3.21 | Block7796-Block7829 | 806509-2413555 |
|  | *qGW-16.1* | Chr16 | 2017 | Block8781 | 24653156 | 2.56 | 6.80 | -1.57 | Block8766-Block8784 | 21243567-25056698 |
|  | *qGW-20.1* | Chr20 | 2017 | Block10718 | 34571040 | 2.82 | 7.40 | 3.21 | Block10704-Block10732 | 33725575-35020508 |

R1, Flowering time; R8, Maturity; RP, Reproductive period; PM, The percentage of RP within the R8; SW, 100-seed weight; GW, Grain weight of per plant.

LOD, log of odds.

PVE, phenotypic variance explained.

Additive effect, (+) Positive additive effects indicate that ZY06 alleles increased the phenotypic value; (-) Negative additive effects indicate that SN14 alleles increased the phenotypic value.

| **Table S4. Chr09-cluster-1 contains candidate genes and coding sequence differences between ZY06 and SN14** | | | | | |
| --- | --- | --- | --- | --- | --- |
| Candidate gene | Annotation | Position | Reference | Alternative | Information |
| *Glyma.09G143500* | *TFL1* | no | no | no | no |
|  |  |  |  |  |  |
| *Glyma.09G149000* | *AGL6* | 38258458 | A | C | synonymous |
|  |  | 38265162 | G | A | nonsynonymous |
|  |  | 38268001 | T | C | synonymous |
|  |  |  |  |  |  |
| *Glyma.09G161300* | *PAF2* | no | no | no | no |
|  |  |  |  |  |  |
| *Glyma.09G185800* | *FLD-like* | 42563465 | G | A | synonymous |
|  |  | 42563519 | G | A | synonymous |
|  |  | 42565329 | A | G | nonsynonymous |
|  |  | 42565355 | A | G | synonymous |
|  |  |  |  |  |  |
| *Glyma.09G187900* | *FTIP* | 42722292 | A | G | nonsynonymous |
|  |  | 42723392 | A | G | synonymous |
|  |  | 42723694 | A | T | nonsynonymous |
|  |  |  |  |  |  |
| *Glyma.09G188000* | *VIN3-L* | 42726027 | A | T | synonymous |
|  |  | 42726390 | G | A | synonymous |
|  |  | 42726816 | G | A | synonymous |
|  |  | 42727095 | T | A | synonymous |
|  |  | 42728400 | C | T | synonymous |

Reference, SN14 coding sequence.

Alternative, ZY06 coding sequence.
